# Biomarkers and Early Mechanisms of Sarcopenia: Central Roles of Mitochondrial Dysfunction, Inflammaging, Cellular Senescence, and Neuromuscular Degeneration

**DOI:** 10.3390/ijms27146332

**Published:** 2026-07-16

**Authors:** Hechmi Toumi, Ahmad Almhdie-Imjabbar, Nada Ibrahim, Eric Lespessailles

**Affiliations:** 1 Translational Medicine Research Platform, PRIMMO, University Hospital Center of Orleans, 14 Avenue de l’Hôpital, 45100 Orléans, France; eric.lespessailles@chu-orleans.fr; 2 Department of Rheumatology, University Hospital Center of Orleans, 14 Avenue de l’Hôpital, 45100 Orléans, France; nada.ibrahim@chu-orleans.fr; 3 Faculty of Sciences and Technology, University of Orleans, 1 Rue de Chartres, 45100 Orléans, France; 4 Laboratory of Metabolic Adaptations to Exercise in Physiological and Pathological Conditions (AME2P), Clermont Auvergne University (UCA), 3 Rue de la Chebarde, 63178 Aubière, France

**Keywords:** sarcopenia, biomarkers, mitochondrial dysfunction, inflammaging, cellular senescence, SASP, denervation, neuromuscular junction, GDF-15, CAF22, IGF-1, myostatin

## Abstract

Sarcopenia is a progressive age-related skeletal muscle disorder characterized by the loss of muscle mass, strength, and physical performance, leading to frailty, disability, and increased mortality. Although its clinical consequences are well recognized, the underlying biological mechanisms remain incompletely understood, limiting the development of early diagnostic strategies and targeted therapies. Increasing evidence indicates that sarcopenia results from complex interactions among mitochondrial dysfunction, chronic low-grade inflammation (inflammaging), cellular senescence, neuromuscular junction degeneration, and anabolic resistance. The present review critically summarizes the current evidence on the principal circulating and molecular biomarkers associated with these interconnected mechanisms. Mitochondrial dysfunction appears to represent an early upstream event that promotes excessive reactive oxygen species production, defective mitophagy, inflammatory activation, and cellular senescence. Chronic inflammation, mediated primarily through IL-6 and TNF-α, further accelerates muscle catabolism and regenerative failure, whereas senescence-associated pathways impair satellite cell function and muscle repair. Neuromuscular degeneration and anabolic resistance further contribute to progressive muscle atrophy and functional decline. Among the candidate biomarkers, GDF-15, FGF-21, IL-6, TNF-α, CAF22, p16INK4a, p21/CDKN1A, IGF-1, and myostatin appear particularly promising for characterizing the biological heterogeneity of sarcopenia. However, no single biomarker currently demonstrates sufficient diagnostic accuracy for routine clinical use. Instead, integrated multi-biomarker approaches combining mitochondrial, inflammatory, senescence-associated, neuromuscular, and anabolic markers may improve early diagnosis, risk stratification, and personalized therapeutic strategies. Future prospective longitudinal studies are required to validate these biomarkers and facilitate their translation into clinical practice.

## 1. Introduction

Sarcopenia is a progressive geriatric syndrome characterized by the decline of skeletal muscle mass, muscle strength, and physical performance, substantially contributing to frailty, falls, disability, loss of independence, and mortality in older adults [1]. Due to the rapid aging of the global population, sarcopenia has become a major public health concern associated with increased hospitalization rates, healthcare costs, and reduced quality of life [2].

Current diagnostic criteria for sarcopenia mainly rely on functional and morphological assessments, including handgrip strength, gait speed, appendicular lean mass, dual-energy X-ray absorptiometry (DXA), and bioelectrical impedance analysis (BIA) [1]. However, these approaches frequently identify sarcopenia only after substantial structural and functional muscle deterioration has already occurred. Consequently, there is growing interest in identifying circulating and molecular biomarkers capable of detecting the early biological alterations underlying sarcopenia pathogenesis [3].

**Figure 1:**
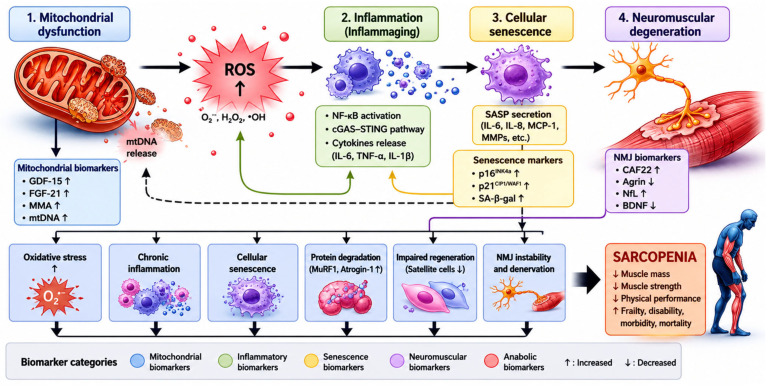
Integrated mechanistic hypothesis of sarcopenia. This figure illustrates the major interconnected biological pathways involved in sarcopenia, highlighting mitochondrial dysfunction as a central mechanism linking inflammaging, cellular senescence, neuromuscular degeneration, anabolic resistance, and progressive muscle loss. Concept conceived and initially sketched by the authors. The graphical illustration was generated using OpenAI ChatGPT (GPT-5) based on the authors’ original sketch and subsequently refined, labeled, and scientifically validated in Microsoft PowerPoint by the authors. Abbreviations: GDF-15: Growth Differentiation Factor-15; FGF-21: Fibroblast Growth Factor-21; MMA: methylmalonic acid; mtDNA: mitochondrial DNA; IL-6: interleukin-6; TNF-α: tumor necrosis factor-alpha; IL-1β: interleukin-1 beta; MCP-1: monocyte chemoattractant protein-1; MMPs: Matrix metalloproteinases; p16^INK4a^: cyclin-dependent kinase inhibitor 2A; p21^CIP/WAF1^: cyclin-dependent kinase inhibitor 1A; SA-β-gal: senescence-associated beta-galactosidase; CAF22: C-terminal agrin fragment; NfL: neurofilament light chain; BDNF: brain-derived neurotrophic factor; ↑: increased/activated; ↓: decreased/inhibited.

The pathophysiology of sarcopenia is highly complex and multifactorial. Several interconnected mechanisms have been implicated, including mitochondrial dysfunction, oxidative stress, chronic low-grade inflammation (“inflammaging”), anabolic resistance, cellular senescence, and neuromuscular degeneration [1,4]. Among these mechanisms, mitochondrial dysfunction has emerged as a potentially central driver because of its critical role in cellular energy production, reactive oxygen species (ROS) generation, redox homeostasis, apoptosis regulation, and inflammatory signaling [4]. Accumulating evidence suggests that mitochondrial impairment promotes excessive ROS production, leading to the activation of inflammatory pathways such as NF-κB and cGAS-STING, increased secretion of pro-inflammatory cytokines including interleukin-6 (IL-6) and tumor necrosis factor-alpha (TNF-α), and induction of muscle cellular senescence [5,6]. These alterations subsequently contribute to impaired muscle regeneration, increased protein degradation, neuromuscular junction instability, and progressive muscle atrophy [4]. Increasing evidence therefore supports the concept of sarcopenia as a systemic and interconnected biological network involving mitochondrial dysfunction, inflammation, cellular senescence, and neuromuscular degeneration [3]. The interactions between these biological mechanisms are illustrated in Figure 1.

Recent studies have identified several promising circulating biomarkers associated with sarcopenia. Mitochondrial-related biomarkers such as Growth Differentiation Factor 15 (GDF-15), Fibroblast Growth Factor 21 (FGF-21), and methylmalonic acid (MMA) have shown strong associations with muscle dysfunction, frailty, and mortality [7]. Recent findings from the Korean Frailty and Aging Cohort Study further demonstrated that combining multiple circulating biomarkers, including GDF-15, FGF-21, P3NP, BDNF, creatinine-based biomarkers, and metabolomic markers, provides higher diagnostic accuracy than individual biomarkers alone [8]. In parallel, inflammatory biomarkers such as Interleukin 6 and Tumor Necrosis Factor alpha, as well as neuromuscular biomarkers including the C-terminal agrin fragment (CAF22) and Neurofilament light chain (NfL), have emerged as potential indicators of muscle degeneration and functional decline [3]. The major categories of biomarkers currently implicated in sarcopenia are summarized in Table 1.

Despite the growing number of proposed biomarkers, no single biomarker currently demonstrates sufficient sensitivity and specificity for independent clinical use. Increasing evidence instead supports the concept of sarcopenia as a systemic and interconnected biological network involving mitochondrial dysfunction, inflammation, cellular senescence, and neuromuscular degeneration [3]. Therefore, the aim of this review is to critically analyze the major biomarkers associated with sarcopenia, with a particular focus on their mechanistic interactions and the early biological events potentially responsible for initiating the sarcopenic cascade. Special attention will be given to the role of mitochondrial dysfunction as a central integrative mechanism linking oxidative stress, inflammaging, cellular senescence, and neuromuscular impairment.

## 2. Methods

### 2.1. Literature Search Strategy

This narrative review was conducted using targeted bibliographic searches performed in the PubMed database to identify recent studies investigating biomarkers and mechanistic pathways involved in sarcopenia pathogenesis. The search strategy specifically focused on mitochondrial dysfunction, cellular senescence, inflammaging, denervation, and neuromuscular junction degeneration as potential early events contributing to skeletal muscle decline.

Four independent thematic searches were conducted using predefined combinations of keywords and Title/Abstract restrictions to improve specificity for mechanistic studies.

#### 2.1.1. Early Pathogenic Mechanisms and Mitochondrial Dysfunction

The first search focused on early pathogenic events associated with mitochondrial dysfunction, denervation, and Growth Differentiation Factor-15 (GDF-15):

****

(sarcopenia[Title/Abstract])

AND

(

“GDF-15”[Title/Abstract]

OR denervation[Title/Abstract]

OR “mitochondrial dysfunction”[Title/Abstract]

)

AND

(

pathogenesis[Title/Abstract]

OR “early events”[Title/Abstract]

)

NOT

(review[Publication Type])

****

This search retrieved 28 articles investigating mitochondrial dysfunction, oxidative stress, denervation pathways, GDF-15 signaling, and early biological mechanisms potentially initiating sarcopenia.

#### 2.1.2. Cellular Senescence and SASP Pathways

The second search investigated cellular senescence-related mechanisms, including senescence-associated secretory phenotype (SASP) signaling and p16INK4a-associated pathways:

****

(sarcopenia[Title/Abstract])

AND

(

senescence[Title/Abstract]

OR SASP[Title/Abstract]

OR p16INK4a[Title/Abstract]

)

AND

(

pathogenesis[Title/Abstract]

OR mechanisms[Title/Abstract]

)

NOT

(review[Publication Type])

****

This search retrieved 83 articles focused on cellular senescence, p16INK4a/p21/CDKN1A signaling, SASP-associated inflammation, stem-cell exhaustion, and impaired regenerative mechanisms involved in sarcopenia progression.

#### 2.1.3. Neuromuscular Junction Degeneration Biomarkers

The third search focused on neuromuscular junction degeneration and related circulating biomarkers, including Agrin and C-terminal agrin fragment (CAF):

****

(sarcopenia[Title/Abstract])

AND

(

CAF[Title/Abstract]

OR agrin[Title/Abstract]

OR “neuromuscular junction”[Title/Abstract]

)

NOT

(review[Publication Type])

****

This search retrieved 161 articles investigating neuromuscular junction degeneration, agrin fragmentation, CAF22, denervation biomarkers, motor neuron dysfunction, and neuromuscular alterations associated with sarcopenia.

#### 2.1.4. Mitochondrial Biomarkers and Metabolic Dysfunction

The fourth search explored mitochondrial biomarkers and metabolic dysfunction associated with skeletal muscle aging:

****

(sarcopenia[Title/Abstract])

AND

(

“GDF-15”[Title/Abstract]

OR “FGF-21”[Title/Abstract]

OR mitochondria[Title/Abstract]

)

NOT

(review[Publication Type])

****

This search retrieved 415 articles focused on mitochondrial biomarkers, including GDF-15 and FGF-21, mitochondrial remodeling, oxidative phosphorylation impairment, reactive oxygen species production, metabolic dysregulation, and inflammaging pathways associated with sarcopenia.

Only English-language original research articles were considered eligible for inclusion. Review articles and meta-analyses were excluded to prioritize mechanistic and translational evidence derived from experimental and clinical studies.

Following title and abstract screening, studies were selected according to their relevance to:

- Early pathogenic mechanisms;
- Mitochondrial dysfunction;
- Inflammaging;
- Cellular senescence;
- Neuromuscular degeneration;
- Biomarker identification in sarcopenia.

**Figure 2:**
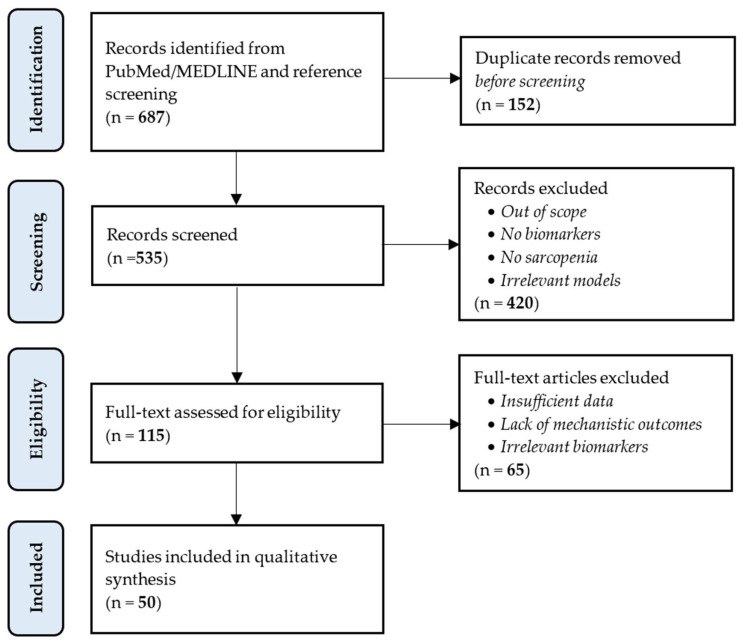
PRISMA-inspired flow diagram of literature selection.

The retrieved studies were subsequently analyzed and integrated according to the principal biological pathways implicated in sarcopenia progression. Particular attention was given to mechanistic interactions linking mitochondrial dysfunction, oxidative stress, inflammatory signaling, senescence pathways, and neuromuscular degeneration. The overall literature selection strategy is summarized in Figure 2.

## 3. Mitochondrial Dysfunction as an Early Driver of Sarcopenia

Mitochondrial dysfunction is increasingly recognized as one of the earliest biological events contributing to sarcopenia development. Skeletal muscle is highly dependent on mitochondrial oxidative phosphorylation for ATP production, calcium homeostasis, redox balance, and maintenance of muscle contractility. Age-related mitochondrial alterations therefore have profound consequences on muscle integrity and function [4,22].

Several studies have demonstrated that aging skeletal muscle exhibits reduced mitochondrial biogenesis, impaired respiratory chain activity, decreased ATP synthesis, and abnormal mitochondrial dynamics characterized by altered fusion and fission processes [5]. These mitochondrial abnormalities promote excessive production of reactive oxygen species (ROS), leading to oxidative damage of proteins, lipids, and mitochondrial DNA (mtDNA) [11]. Persistent oxidative stress subsequently activates catabolic signaling pathways and contributes to progressive muscle degeneration.

Recent evidence suggests that mitochondrial dysfunction may act upstream of inflammation and cellular senescence in sarcopenia pathogenesis. Damaged mitochondria release mitochondrial DNA fragments and danger-associated molecular patterns (DAMPs), which activate inflammatory pathways including NF-κB and cGAS-STING signaling [5]. Recent transcriptomic analyses have further identified several cuproptosis-related genes (PDHA1, DLAT, PDHB, and NDUFC1) as potential diagnostic biomarkers of sarcopenia, reinforcing the central role of mitochondrial energy metabolism in disease pathogenesis [23].

Activation of these pathways promotes chronic low-grade inflammation (“inflammaging”) and enhances secretion of pro-inflammatory cytokines such as IL-6 and TNF-α, both strongly associated with muscle wasting and physical decline [12].

Mitochondrial dysfunction is also closely linked to impaired autophagy and defective mitophagy. Under physiological conditions, damaged mitochondria are selectively removed through mitophagy to preserve mitochondrial quality control. However, aging muscle demonstrates progressive impairment of autophagic flux, leading to accumulation of dysfunctional mitochondria and amplification of oxidative stress [24]. This vicious cycle further accelerates muscle atrophy and contractile dysfunction.

Among the emerging circulating biomarkers of mitochondrial stress, Growth Differentiation Factor 15 (GDF-15) has received increasing attention. GDF-15 is a stress-responsive cytokine induced by mitochondrial injury, oxidative stress, and inflammation. Elevated circulating GDF-15 levels have been associated with frailty, reduced muscle strength, poor physical performance, and mortality in older adults [9].

Recent mechanistic studies further suggest that GDF-15 may reflect early mitochondrial impairment preceding overt muscle mass decline. Recent population-based evidence from the SardiNIA cohort further confirmed that circulating GDF-15 levels are independently associated with reduced handgrip strength and impaired physical performance, reinforcing its potential clinical value as a biomarker of sarcopenia [25].

Another important mitochondrial biomarker is Fibroblast Growth Factor 21 (FGF-21), a metabolic regulator induced under conditions of mitochondrial stress and energetic imbalance. Increased FGF-21 expression has been observed in aging skeletal muscle and may represent a compensatory response to impaired oxidative metabolism [10]. Elevated FGF-21 concentrations have also been linked to reduced muscle quality and physical dysfunction in elderly individuals.

Circulating mitochondrial transcription factor A has recently been associated with poorer physical function and greater sarcopenia risk in community-dwelling older adults, particularly in women, suggesting that TFAM may represent an additional circulating marker of mitochondrial stress and functional impairment [26].

Altered expression of genes involved in mitochondrial biogenesis, dynamics, and quality control has been associated with impaired muscle bioenergetics and skeletal muscle aging, particularly pathways involving PGC-1α-related mitochondrial biogenesis and OPA1-dependent mitochondrial dynamics [5,10].

Metabolomic studies have also identified methylmalonic acid (MMA) as a potential indicator of mitochondrial metabolic dysfunction in sarcopenic patients.

Importantly, mitochondrial dysfunction may also contribute to neuromuscular degeneration. Reduced ATP availability and increased oxidative stress impair neuromuscular junction maintenance, promote motor neuron instability, and facilitate denervation processes associated with muscle weakness [18]. This interaction suggests that mitochondrial impairment may constitute a central mechanistic hub linking inflammation, senescence, and neuromuscular degeneration during sarcopenia progression.

Collectively, these findings support the hypothesis that mitochondrial dysfunction represents a major early driver of sarcopenia and a potential upstream mechanism integrating oxidative stress, inflammaging, cellular senescence, and denervation pathways.

**Figure 3:**
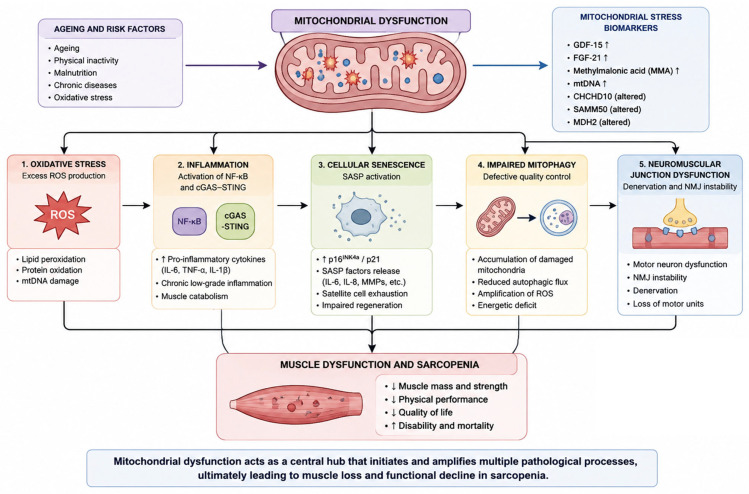
Mitochondrial dysfunction as a central mechanistic hub in sarcopenia. This figure illustrates how impaired mitochondrial homeostasis promotes oxidative stress, defective mitophagy, chronic inflammation, cellular senescence, and skeletal muscle atrophy. Concept conceived and initially sketched by the authors. Graphical rendering generated using OpenAI ChatGPT (GPT-5) and finalized in Microsoft PowerPoint by the authors. Abbreviations: GDF-15: Growth Differentiation Factor-15; FGF-21: Fibroblast Growth Factor-21; MMA: methylmalonic acid; mtDNA: mitochondrial DNA; CHCHD10: coiled-coil-helix-coiled-coil-helix domain containing 10; SAMM50: sorting and assembly machinery component 50 homolog; MDH2: malate dehydrogenase 2; ROS: reactive oxygen species; NF-κB: nuclear factor kappa B; cGAS: cyclic GMP-AMP synthase; IL-6: interleukin-6; TNF-α: tumor necrosis factor-alpha; IL-1β: interleukin-1 beta; p16^INK4a^: cyclin-dependent kinase inhibitor 2A; p21: cyclin-dependent kinase inhibitor 1A; SASP: senescence-associated secretory phenotype; MMPs: Matrix metalloproteinases; ↑: increased/activated; ↓: decreased/inhibited.

The principal mitochondrial biomarkers currently associated with sarcopenia, their biological functions, and their clinical relevance are summarized in Table 2, and the molecular interactions linking mitochondrial dysfunction to downstream pathological mechanisms involved in sarcopenia are summarized in Figure 3.

## 4. Inflammaging and Chronic Low-Grade Inflammation in Sarcopenia

Chronic low-grade inflammation, commonly referred to as “inflammaging,” is considered a major contributor to the development and progression of sarcopenia. Aging is associated with persistent activation of inflammatory signaling pathways, resulting in elevated circulating concentrations of pro-inflammatory cytokines that negatively affect skeletal muscle homeostasis [31].

Among the inflammatory mediators implicated in sarcopenia, Interleukin 6 (IL-6) and Tumor Necrosis Factor alpha (TNF-α) are the most extensively studied. Elevated circulating IL-6 and TNF-α levels have consistently been associated with reduced muscle mass, decreased muscle strength, impaired physical performance, frailty, and increased mortality in older adults [12,13]. Recent clinical evidence further indicates that a combination of inflammatory cytokines, including IL-6, TNF-α, IL-17A and IL-10, discriminates sarcopenic from non-sarcopenic older adults more effectively than individual biomarkers, supporting the concept of multi-biomarker diagnostic strategies [32]. Chronic exposure to these cytokines promotes muscle protein degradation through activation of catabolic pathways including the ubiquitin–proteasome system and NF-κB signaling. Population-based NHANES data further suggest that the neutrophil percentage-to-albumin ratio, an easily accessible marker combining inflammation and nutritional status, is independently and nonlinearly associated with sarcopenia risk and improves prediction beyond traditional risk factors [33].

NF-κB plays a central role in inflammation-induced muscle wasting. Under physiological conditions, NF-κB activation is transient and tightly regulated. However, aging-related oxidative stress and mitochondrial dysfunction induce persistent NF-κB activation, leading to sustained expression of inflammatory cytokines, muscle catabolism, and impaired regenerative capacity [21]. Importantly, mitochondrial reactive oxygen species (ROS) amplify NF-κB signaling, creating a vicious cycle linking mitochondrial dysfunction and chronic inflammation.

Recent findings have also highlighted the importance of the cGAS-STING pathway in sarcopenia-related inflammaging. Mitochondrial damage results in leakage of mitochondrial DNA (mtDNA) into the cytosol, where it activates cyclic GMP–AMP synthase (cGAS) and stimulator of interferon genes (STING), triggering inflammatory responses and type I interferon signaling [5].

Persistent activation of this pathway contributes to chronic inflammation, cellular senescence, and muscle degeneration.

Inflammaging also affects muscle regeneration by impairing satellite cell function. Chronic inflammatory exposure reduces the proliferative and regenerative capacity of muscle stem cells while promoting fibrosis and fatty infiltration of skeletal muscle [14]. Inflammatory cytokines further interfere with anabolic signaling pathways such as IGF-1/Akt/mTOR, thereby contributing to anabolic resistance in aging muscle.

In addition to IL-6 and TNF-α, elevated levels of C-reactive protein (CRP) have been associated with sarcopenia severity and physical decline. CRP is considered a systemic marker of chronic inflammation and may reflect the cumulative inflammatory burden associated with aging and multimorbidity.

Importantly, inflammation does not act independently but interacts closely with mitochondrial dysfunction and cellular senescence. Oxidative stress-induced inflammatory signaling promotes senescence-associated secretory phenotype (SASP) activation, whereas senescent cells further release pro-inflammatory mediators that perpetuate inflammaging [15]. This reciprocal interaction may represent a central mechanism driving progressive muscle degeneration.

Collectively, current evidence supports chronic inflammation as a major biological component of sarcopenia pathogenesis. Persistent activation of inflammatory pathways contributes to muscle catabolism, regenerative failure, mitochondrial impairment, and neuromuscular degeneration, thereby accelerating functional decline during aging.

**Figure 4:**
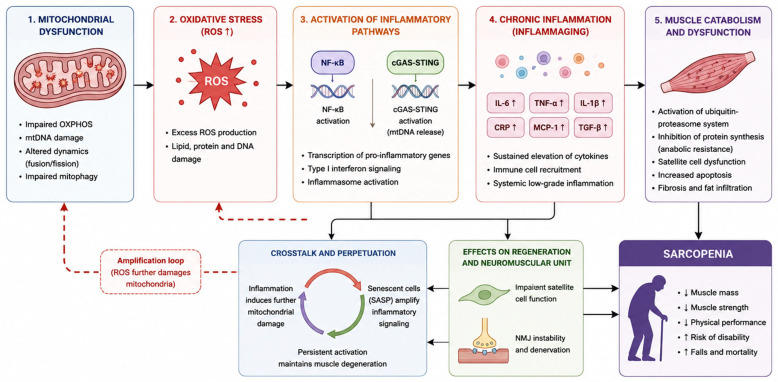
Interactions between mitochondrial dysfunction, oxidative stress, and inflammaging in sarcopenia. This figure illustrates the reciprocal interactions between mitochondrial dysfunction, reactive oxygen species production, inflammatory signaling, and progressive muscle degeneration. Concept conceived and initially sketched by the authors. Graphical rendering generated using OpenAI ChatGPT (GPT-5) and finalized in Microsoft PowerPoint by the authors. Abbreviations: IL-6: interleukin-6; TNF-α: tumor necrosis factor-alpha; IL-1β: interleukin-1 beta; CRP: C-reactive protein; MCP-1: monocyte chemoattractant protein-1; TGF-β: transforming growth factor-beta; SASP: Senescence-associated secretory phenotype; NMJ: Neuromuscular junction; ↑: increased/activated; ↓: decreased/inhibited.

The interactions between oxidative stress, mitochondrial dysfunction, and inflammatory signaling pathways involved in sarcopenia are summarized in Figure 4. Key inflammatory biomarkers associated with sarcopenia and their biological effects are summarized in Table 3.

## 5. Cellular Senescence and SASP Signaling in Sarcopenia

Cellular senescence is increasingly recognized as a major biological mechanism contributing to skeletal muscle aging and sarcopenia progression. Senescence is characterized by irreversible cell-cycle arrest accompanied by profound metabolic and secretory alterations that negatively affect tissue homeostasis [34]. In aging skeletal muscle, the accumulation of senescent cells contributes to impaired regenerative capacity, chronic inflammation, and progressive muscle degeneration.

One of the principal characteristics of senescent cells is the activation of the senescence-associated secretory phenotype (SASP), which includes the secretion of pro-inflammatory cytokines, chemokines, growth factors, and matrix-remodeling enzymes [35]. SASP factors such as IL-6, IL-1β, TNF-α, and TGF-β create a pro-inflammatory microenvironment that amplifies inflammaging and promotes muscle catabolism.

Elevated p16INK4a and p21/CDKN1A expression has been associated with impaired satellite cell activation, reduced regenerative potential, and muscle fiber atrophy in aging skeletal muscle. Satellite cells play a crucial role in muscle regeneration and maintenance. Under normal conditions, these stem cells remain quiescent until activated in response to muscle injury or stress. However, aging-associated senescence progressively impairs satellite cell self-renewal and differentiation capacity [14]. Senescent satellite cells exhibit mitochondrial dysfunction, oxidative stress accumulation, altered autophagy, and reduced regenerative efficiency, ultimately contributing to muscle mass decline.

Mitochondrial dysfunction and oxidative stress are closely interconnected with senescence pathways. Excessive reactive oxygen species (ROS) production induces DNA damage responses and activates p53/p21/CDKN1A signaling, thereby promoting senescence induction [6]. Conversely, senescent cells further exacerbate mitochondrial dysfunction through persistent inflammatory signaling and impaired mitophagy, creating a self-amplifying pathological cycle.

Emerging evidence also suggests that the cGAS-STING pathway contributes to senescence-associated inflammation in sarcopenia. Cytosolic accumulation of damaged mitochondrial DNA activates innate immune signaling, resulting in increased SASP production and chronic inflammatory activation [5].

This interaction further strengthens the mechanistic link between mitochondrial dysfunction, inflammaging, and cellular senescence.

In addition to inflammatory amplification, senescence contributes directly to anabolic resistance and muscle wasting. SASP-associated cytokines inhibit IGF-1/Akt/mTOR signaling, impair protein synthesis, and stimulate proteolytic pathways such as ubiquitin–proteasome and autophagy-lysosome systems. Persistent senescence signaling therefore accelerates muscle degeneration and functional decline.

Several senescence-associated biomarkers are currently being investigated as potential indicators of sarcopenia severity and progression. These include p16INK4a, p21/CDKN1A, senescence-associated β-galactosidase (SA-β-gal), SASP cytokines, and DNA damage markers such as γH2AX. However, none of these biomarkers alone currently possesses sufficient specificity for clinical diagnosis.

Importantly, recent experimental studies suggest that targeting senescent cells through senolytic therapies may partially restore muscle regeneration and improve physical function in aging models [16]. These findings support the concept that cellular senescence is not only a biomarker source but also a potentially reversible therapeutic target in sarcopenia.

Collectively, current evidence indicates that cellular senescence acts as a central biological amplifier linking mitochondrial dysfunction, chronic inflammation, stem-cell exhaustion, and impaired muscle regeneration during sarcopenia progression.

**Figure 5:**
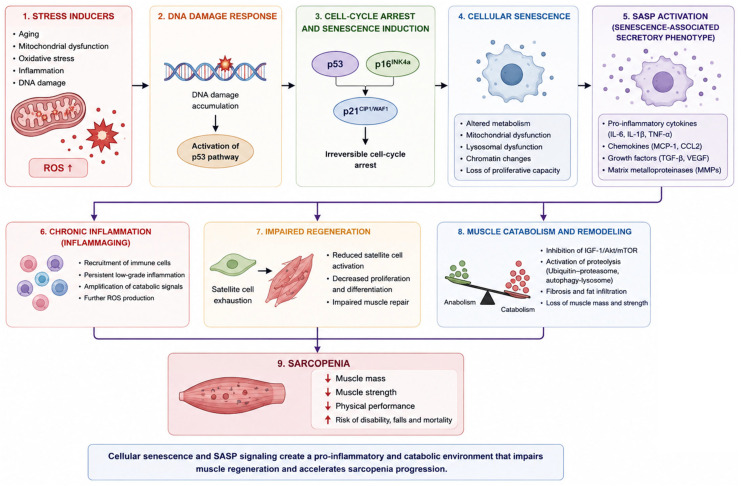
Cellular senescence and SASP signaling pathways in sarcopenia. This figure illustrates how cellular senescence contributes to sarcopenia through activation of the p16INK4a and p21/CDKN1A pathways, leading to the development of the senescence-associated secretory phenotype (SASP), chronic inflammation, impaired muscle regeneration, and progressive muscle atrophy. Concept conceived and initially sketched by the authors. Graphical rendering generated using OpenAI ChatGPT (GPT-5) and finalized in Microsoft PowerPoint by the authors. Abbreviations: SASP: Senescence-associated secretory phenotype; IL-1β: interleukin-1 beta; TNF-α: tumor necrosis factor-alpha; TGF-β: transforming growth factor-beta; MCP-1: monocyte chemoattractant protein-1; MMPs: Matrix metalloproteinases; IGF-1: Insulin-like growth factor 1; Akt: Protein kinase B; mTOR: Mammalian target of rapamycin; ↑: increased/activated; ↓: decreased/inhibited.

The major senescence-associated biomarkers identified in sarcopenia and their biological implications are summarized in Table 4. The principal senescence pathways involved in sarcopenia are summarized in Figure 5.

## 6. Neuromuscular Junction Degeneration and Denervation in Sarcopenia

Neuromuscular junction (NMJ) degeneration is increasingly recognized as a critical contributor to sarcopenia progression. The NMJ is a highly specialized synapse responsible for transmitting signals from motor neurons to skeletal muscle fibers, thereby ensuring muscle contraction and motor function. Aging-related alterations in NMJ integrity contribute to denervation, motor unit loss, impaired muscle activation, and progressive skeletal muscle atrophy [18].

Several studies suggest that neuromuscular degeneration may occur early during sarcopenia development, potentially preceding measurable reductions in muscle mass. Age-associated degeneration of motor neurons results in partial or complete denervation of muscle fibers, leading to impaired muscle function, fiber atrophy, and reduced regenerative capacity [4]. Although compensatory reinnervation may initially occur, this process becomes progressively insufficient with aging, ultimately resulting in irreversible motor unit loss.

Mitochondrial dysfunction and oxidative stress play major roles in NMJ degeneration. Motor neurons and NMJ structures are highly dependent on mitochondrial ATP production to maintain synaptic transmission and axonal integrity. Excessive reactive oxygen species (ROS) production damages synaptic proteins, impairs acetylcholine receptor clustering, and destabilizes NMJ architecture [18]. Chronic oxidative stress also promotes axonal degeneration and neuromuscular transmission failure.

Among the most studied circulating biomarkers of NMJ degeneration is the C-terminal agrin fragment (CAF22), a cleavage product generated by agrin degradation. Agrin is an essential synaptic protein involved in acetylcholine receptor stabilization and NMJ maintenance. CAF22 has emerged as one of the most promising circulating biomarkers reflecting neuromuscular junction degeneration. Initial evidence demonstrated that elevated circulating CAF22 levels were associated with sarcopenia caused by neuromuscular junction degeneration [17]. This observation was subsequently confirmed in the prospective ilSIRENTE cohort, where higher serum CAF concentrations were independently associated with sarcopenia after adjustment for age, comorbidities, inflammatory markers, and functional status [36]. More recent clinical evidence also supports the relevance of agrin-related biomarkers, showing that circulating agrin alterations are associated with muscle strength decline and reduced expression of agrin receptors involved in neuromuscular junction integrity [37]. Increased circulating CAF22 levels have been associated with NMJ destabilization, reduced muscle strength, impaired physical performance, and frailty in older adults [17]. Elevated CAF22 concentrations are therefore considered potential indicators of denervation and neuromuscular dysfunction in sarcopenia.

Another important NMJ-related biomarker is Agrin itself. Reduced agrin expression contributes directly to synaptic instability and impaired neuromuscular transmission. Experimental studies have demonstrated that agrin deficiency accelerates muscle wasting and NMJ fragmentation during aging.

Recent evidence has also highlighted the potential role of Neurofilament light chain (NfL) as a biomarker of motor neuron injury and axonal degeneration in sarcopenia. Elevated circulating NfL levels reflect neuroaxonal damage and have been associated with physical decline and neuromuscular impairment in elderly individuals.

Brain-derived neurotrophic factor (BDNF) has additionally emerged as a regulator of neuromuscular maintenance and muscle regeneration. Reduced BDNF signaling may impair motor neuron survival and satellite cell activation, thereby contributing to NMJ degeneration and muscle dysfunction. Recent evidence in 646 older adults showed that lower plasma BDNF levels were associated with sarcopenia, lower handgrip strength, slower gait speed, and impaired wearable sensor-derived gait parameters, supporting BDNF as a biomarker of the brain–muscle axis in sarcopenia [38].

Importantly, NMJ degeneration interacts closely with inflammation and cellular senescence. Chronic inflammatory cytokines such as IL-6 and TNF-α impair motor neuron survival and accelerate synaptic degeneration. Similarly, senescence-associated secretory phenotype (SASP) factors may contribute to progressive denervation through persistent inflammatory activation and tissue remodeling.

Emerging evidence suggests that denervation may represent one of the earliest detectable functional events during sarcopenia progression. Several longitudinal studies indicate that muscle strength declines more rapidly than muscle mass, supporting the hypothesis that neuromuscular dysfunction may precede overt muscle atrophy [19]. This observation further strengthens the importance of NMJ biomarkers for early diagnosis and risk stratification.

Collectively, these findings support the concept that neuromuscular degeneration represents a major mechanistic component of sarcopenia, interacting closely with mitochondrial dysfunction, inflammaging, and senescence pathways to promote progressive muscle weakness and functional decline.

**Figure 6:**
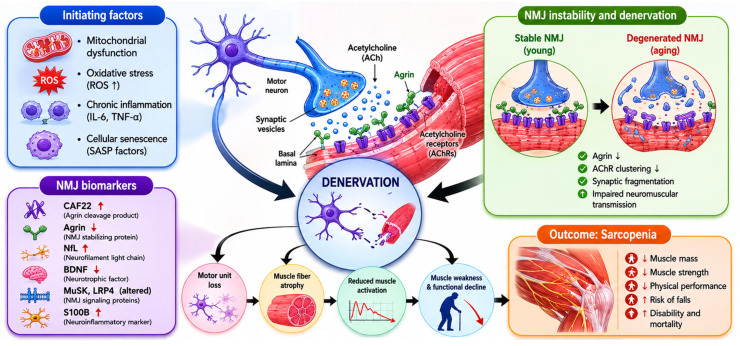
Neuromuscular junction degeneration and denervation pathways in sarcopenia. This figure illustrates the mechanisms of age-related neuromuscular junction degeneration, motor neuron dysfunction, denervation, and their contribution to muscle weakness and functional decline, together with the principal neuromuscular biomarkers. Concept conceived and initially sketched by the authors. Graphical rendering generated using OpenAI ChatGPT (GPT-5) and finalized in Microsoft PowerPoint by the authors. Abbreviations: ROS: reactive oxygen species; IL-6: interleukin-6; TNF-α: tumor necrosis factor-alpha; CAF22: C-terminal agrin fragment; NfL: neurofilament light chain; BDNF: brain-derived neurotrophic factor; MuSK: muscle-specific kinase; LRP4: low-density lipoprotein receptor-related protein 4; ACh: acetylcholine; AChRs: acetylcholine receptors; ↑: increased/activated; ↓: decreased/inhibited.

The main biomarkers reflecting neuromuscular junction degeneration and motor-unit remodeling are presented in Table 5. The principal mechanisms linking NMJ degeneration and denervation to sarcopenia are summarized in Figure 6.

## 7. Anabolic Resistance and Muscle Remodeling in Sarcopenia

Anabolic resistance is a hallmark of aging skeletal muscle and represents a major contributor to sarcopenia progression. It is characterized by a reduced ability of skeletal muscle to respond adequately to anabolic stimuli such as dietary protein intake, amino acids, insulin, and physical exercise [20]. This impaired anabolic responsiveness results in progressive imbalance between protein synthesis and protein degradation, ultimately leading to muscle atrophy and functional decline.

One of the central anabolic pathways involved in muscle homeostasis is the insulin-like growth factor-1 (IGF-1)/Akt/mTOR signaling pathway. Under physiological conditions, Insulin-like Growth Factor 1 (IGF-1) stimulates protein synthesis, satellite cell activation, and muscle regeneration through activation of Akt and mammalian target of rapamycin (mTOR) signaling [39]. However, aging is associated with reduced circulating IGF-1 levels and impaired downstream anabolic signaling, contributing to decreased muscle protein synthesis and regenerative failure.

In parallel, aging muscle demonstrates increased activation of catabolic pathways. Among the most important catabolic regulators is Myostatin, a member of the transforming growth factor-beta (TGF-β) superfamily that negatively regulates muscle growth. Elevated myostatin expression inhibits myoblast proliferation, suppresses protein synthesis, and stimulates muscle proteolysis through activation of ubiquitin–proteasome pathways [40]. Increased myostatin levels have consistently been associated with reduced muscle mass and impaired physical performance in sarcopenic individuals.

The ubiquitin–proteasome system represents a major proteolytic mechanism involved in muscle wasting. Muscle-specific E3 ubiquitin ligases such as MuRF1 and Atrogin-1 are significantly upregulated during muscle atrophy and contribute to accelerated degradation of contractile proteins [41]. Chronic inflammation and oxidative stress further amplify activation of these catabolic pathways.

Mitochondrial dysfunction and cellular senescence also contribute substantially to anabolic resistance. Reduced mitochondrial ATP production impairs energy availability for protein synthesis, whereas senescence-associated secretory phenotype (SASP) factors interfere with IGF-1/Akt/mTOR signaling and promote chronic catabolic activation. Persistent inflammatory signaling therefore creates a metabolic environment favoring muscle degradation rather than regeneration.

Satellite cell dysfunction additionally plays an important role in impaired muscle remodeling. Aging-associated reductions in satellite cell number and regenerative potential limit the ability of skeletal muscle to repair damaged fibers and maintain muscle mass [14]. Fibrosis and adipose tissue infiltration further impair muscle quality and contractile efficiency.

Several biomarkers associated with anabolic resistance and muscle remodeling have emerged as potential indicators of sarcopenia severity. Reduced circulating IGF-1 levels are frequently associated with decreased muscle mass and impaired functional performance. Conversely, elevated myostatin concentrations correlate with muscle wasting and physical decline.

Other emerging biomarkers include procollagen type III N-terminal peptide (P3NP), a marker of muscle remodeling and collagen turnover, and SPARC (secreted protein acidic and rich in cysteine), a matricellular glycoprotein involved in extracellular matrix remodeling and muscle adaptation. Altered SPARC expression has been associated with impaired muscle regeneration and aging-related remodeling defects.

Importantly, anabolic resistance is potentially reversible through interventions such as resistance exercise, protein supplementation, mitochondrial-targeted therapies, and anti-inflammatory approaches. Resistance training remains one of the most effective interventions for improving anabolic sensitivity and stimulating muscle protein synthesis in older adults.

Collectively, current evidence indicates that anabolic resistance results from complex interactions between impaired anabolic signaling, chronic inflammation, mitochondrial dysfunction, cellular senescence, and proteolytic activation. These mechanisms collectively contribute to progressive muscle wasting and sarcopenia development.

Although numerous biomarkers have been implicated in anabolic resistance and muscle remodeling, their level of clinical validation varies considerably. Circulating biomarkers such as IGF-1 and myostatin have been evaluated in multiple human sarcopenia cohorts and have demonstrated significant associations with muscle mass, muscle strength, and physical performance. P3NP has also shown potential as a marker of muscle remodeling, although clinical evidence remains relatively limited. In contrast, components of the Akt/mTOR, FOXO, MuRF1, and Atrogin-1 signaling pathways, as well as SPARC, are currently supported predominantly by mechanistic and experimental studies conducted in animal models or skeletal muscle tissue. Although these biomarkers provide important insights into the biological mechanisms underlying anabolic resistance, additional prospective studies are required to establish their diagnostic and prognostic value in human sarcopenia.

**Figure 7:**
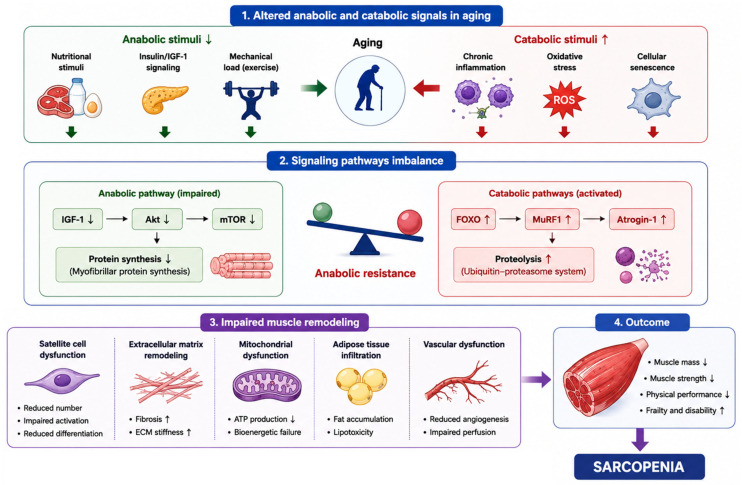
Mechanisms of anabolic resistance and muscle remodeling in sarcopenia. This figure illustrates the imbalance between anabolic and catabolic signaling pathways in sarcopenia, highlighting impaired IGF-1/Akt/mTOR signaling, activation of FOXO, MuRF1, and Atrogin-1, reduced protein synthesis, and progressive muscle wasting. Concept conceived and initially sketched by the authors. Graphical rendering generated using OpenAI ChatGPT (GPT-5) and finalized in Microsoft PowerPoint by the authors. Abbreviations: Akt: protein kinase B; mTOR: mammalian target of rapamycin; FOXO: forkhead box O transcription factors; MuRF1: muscle RING-finger protein-1; Atrogin-1: muscle atrophy F-box protein; ECM: extracellular matrix; ROS: reactive oxygen species; ↑: increased/activated; ↓: decreased/inhibited.

The principal biomarkers involved in anabolic signaling, proteolysis, and muscle remodeling are summarized in Table 6. The principal mechanisms involved in anabolic resistance and muscle remodeling are summarized in Figure 7.

## 8. Integrative Mechanisms and Cross-Talk Between Biomarkers in Sarcopenia

Sarcopenia is increasingly recognized as a multifactorial and interconnected biological process rather than the consequence of a single isolated mechanism. Emerging evidence indicates that mitochondrial dysfunction, inflammaging, cellular senescence, neuromuscular degeneration, and anabolic resistance interact through complex molecular networks that collectively drive progressive skeletal muscle decline [4].

Among these interconnected pathways, mitochondrial dysfunction appears to occupy a central upstream position. Aging-associated mitochondrial impairment leads to reduced ATP production, excessive reactive oxygen species (ROS) generation, altered mitochondrial dynamics, and defective mitophagy. These alterations initiate a cascade of downstream pathological events involving inflammatory activation, senescence induction, and neuromuscular degeneration.

One of the most important links between mitochondrial dysfunction and inflammation involves activation of NF-κB and cGAS-STING signaling pathways. Damaged mitochondria release mitochondrial DNA (mtDNA) and danger-associated molecular patterns (DAMPs), which stimulate innate immune responses and promote chronic low-grade inflammation [5].

Persistent inflammatory activation subsequently enhances secretion of IL-6, TNF-α, and other catabolic cytokines that impair muscle protein synthesis and stimulate proteolysis.

Inflammation and senescence are also tightly interconnected. Chronic inflammatory signaling induces DNA damage and oxidative stress, thereby activating p53/p21/CDKN1A and p16INK4a senescence pathways [15]. In turn, senescent cells release senescence-associated secretory phenotype (SASP) factors that perpetuate inflammation, creating a self-amplifying cycle of inflammaging and tissue degeneration.

Mitochondrial dysfunction additionally contributes directly to anabolic resistance. Reduced mitochondrial oxidative phosphorylation decreases energy availability for protein synthesis, while inflammatory cytokines inhibit IGF-1/Akt/mTOR signaling pathways. Simultaneously, activation of FOXO transcription factors and ubiquitin–proteasome systems promotes muscle protein degradation. These interactions ultimately shift muscle metabolism toward a catabolic state.

Neuromuscular degeneration represents another major component of this interconnected network. Oxidative stress and inflammatory signaling impair neuromuscular junction (NMJ) integrity, leading to denervation and motor unit loss. Elevated levels of CAF22 and neurofilament light chain (NfL) further reflect ongoing neuromuscular degeneration and axonal injury. Importantly, denervation itself accelerates mitochondrial dysfunction and muscle atrophy, establishing additional pathological feedback loops.

Recent longitudinal studies suggest that these biological mechanisms do not occur sequentially but rather evolve simultaneously through reciprocal interactions. For example, mitochondrial dysfunction promotes inflammation and senescence, whereas inflammatory cytokines further aggravate mitochondrial damage and impair mitophagy. Similarly, denervation enhances oxidative stress and catabolic signaling, while anabolic resistance limits compensatory muscle regeneration.

This integrated network perspective may explain why no single biomarker currently demonstrates sufficient diagnostic sensitivity or specificity in sarcopenia. Instead, combined biomarker panels integrating mitochondrial, inflammatory, senescence-associated, and neuromuscular markers may provide superior predictive and diagnostic performance.

Several studies have therefore proposed multi-biomarker approaches combining GDF-15, IL-6, TNF-α, CAF22, IGF-1, and senescence-associated markers to better characterize sarcopenia severity and progression [3]. These integrative strategies may improve early diagnosis, risk stratification, and personalized therapeutic interventions.

Importantly, understanding the molecular cross-talk between these pathways also has therapeutic implications. Targeting mitochondrial dysfunction, reducing chronic inflammation, eliminating senescent cells, and preserving neuromuscular junction integrity may collectively provide more effective approaches than single-target interventions alone.

Collectively, current evidence supports the concept that sarcopenia is a systemic network disease involving dynamic interactions between mitochondrial dysfunction, inflammaging, senescence, denervation, and anabolic resistance. This integrative framework may help explain the heterogeneity of sarcopenia phenotypes and facilitate development of multi-target therapeutic strategies.

**Figure 8:**
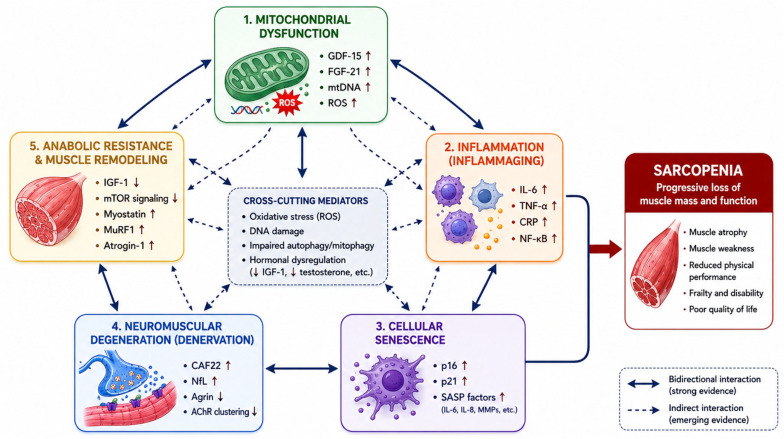
Integrated biomarker interaction network in sarcopenia. This figure illustrates the interactions among mitochondrial dysfunction, inflammaging, cellular senescence, neuromuscular degeneration, and anabolic resistance, emphasizing their collective contribution to the development and progression of sarcopenia. Concept conceived and initially sketched by the authors. Graphical rendering generated using OpenAI ChatGPT (GPT-5) and finalized in Microsoft PowerPoint by the authors. Abbreviations: GDF-15: growth differentiation factor 15; FGF-21: fibroblast growth factor 21; mtDNA: mitochondrial DNA; ROS: reactive oxygen species; IL-6: interleukin-6; TNF-α: tumor necrosis factor-alpha; CRP: C-reactive protein; NF-κB: nuclear factor kappa B; IGF-1: insulin-like growth factor 1; mTOR: mammalian target of rapamycin; MuRF1: muscle RING-finger protein-1; CAF22: C-terminal agrin fragment; NfL: neurofilament light chain; Agrin: agrin protein; AChR: acetylcholine receptor; SASP: senescence-associated secretory phenotype; MMPs: matrix metalloproteinases; ↑: increased/activated; ↓: decreased/inhibited.

The major mechanistic interactions linking these biological pathways are summarized in Table 7. The principal interactions between the major biomarker pathways involved in sarcopenia are summarized in Figure 8.

## 9. Clinical Implications and Future Perspectives of Biomarkers in Sarcopenia

The identification of reliable biomarkers for sarcopenia has become a major priority in aging research and geriatric medicine. Current diagnostic approaches primarily rely on measurements of muscle mass, muscle strength, and physical performance, including handgrip strength, gait speed, and imaging-based assessments [1]. However, these approaches generally detect sarcopenia after significant functional decline has already occurred. Consequently, there is growing interest in identifying early biological markers capable of predicting disease onset before irreversible muscle degeneration develops.

One of the major challenges in sarcopenia biomarker research is the heterogeneity of the disease. Sarcopenia results from complex interactions between aging, inflammation, mitochondrial dysfunction, hormonal changes, denervation, nutritional status, and physical inactivity. As a consequence, no single biomarker currently demonstrates sufficient sensitivity or specificity for universal clinical application. Recent meta-analytic evidence further confirms the heterogeneous diagnostic performance of blood biomarkers for sarcopenia, with creatinine–cystatin C ratio, myostatin, and irisin showing moderate diagnostic accuracy, but no single circulating biomarker demonstrating sufficient performance for universal clinical use [42].

Among the emerging circulating biomarkers, Growth Differentiation Factor 15 (GDF-15), Interleukin 6 (IL-6), Tumor Necrosis Factor alpha (TNF-α), and C-terminal agrin fragment (CAF22) appear particularly promising because they reflect multiple interconnected mechanisms involved in sarcopenia progression. Elevated levels of these biomarkers have consistently been associated with frailty, reduced muscle strength, impaired mobility, and increased mortality risk in older adults. Importantly, the clinical utility of circulating biomarkers may extend beyond the diagnosis of age-related sarcopenia. Growing evidence in patients undergoing radical cystectomy for bladder cancer demonstrated that elevated circulating IL-6 and GDF-15 were significantly associated with sarcopenia and independently predicted poorer overall and cancer-specific survival. These findings suggest that inflammatory and mitochondrial-related biomarkers may also provide valuable prognostic information in disease-associated muscle wasting and support their potential use across diverse clinical settings [43].

Recent evidence suggests that multi-biomarker panels may provide superior diagnostic performance compared with isolated biomarkers. Accumulating evidence from the Korean Frailty and Aging Cohort Study further supports this approach, showing that combined biomarker strategies may outperform isolated circulating biomarkers for sarcopenia diagnosis and phenotyping [8]. Integrative approaches combining mitochondrial biomarkers (GDF-15, FGF-21), inflammatory mediators (IL-6, CRP, TNF-α), senescence markers (p16INK4a, p21/CDKN1A, SASP factors), and neuromuscular biomarkers (CAF22, NfL) may improve early detection and risk stratification of sarcopenic individuals [3]. Emerging evidence from the FRASNET study showed that circulating mitokines GDF-15 and FGF-21 are associated with frailty, sarcopenia, and malnutrition in older adults, supporting their complementary value within integrated biomarker panels [44]. However, not all multi-biomarker strategies demonstrate sufficient diagnostic performance, as an exploratory panel including fecal calprotectin, BDNF, FGF-21, and irisin showed only modest accuracy for probable sarcopenia, highlighting the need for external validation and clinically meaningful thresholds [45].

Advances in omics technologies are also transforming sarcopenia research. Transcriptomics, proteomics, metabolomics, and lipidomics now allow identification of complex molecular signatures associated with muscle aging. Metabolomic studies have identified alterations in amino acid metabolism, mitochondrial intermediates, and lipid metabolism pathways that may precede overt muscle atrophy. Similarly, transcriptomic analyses have identified mitochondrial and inflammatory gene networks associated with progressive functional decline. Metabolomics-based approaches have also identified traumatic acid as a potential plasma biomarker for sarcopenia, supporting the value of metabolic profiling for discovering novel diagnostic signatures beyond conventional inflammatory and mitochondrial markers [46]. Integrated transcriptomic, proteomic, and metabolomic analyses have recently identified circulating immunometabolic markers including CD14, FCGR2A, and LRG1 as candidate biomarkers associated with sarcopenia and muscle strength, illustrating the value of multi-omics approaches for biomarker discovery [47]. Targeted metabolomic profiling has also identified altered bile acid metabolism in older adults with sarcopenia, with elevated 3β-hyodeoxycholic acid and glycolithocholic acid proposed as candidate metabolic biomarkers associated with sarcopenia risk and gait performance [48]. Circulating kynurenine, a tryptophan-derived metabolite linked to oxidative and inflammatory muscle catabolism, has recently been associated with lower skeletal muscle index, weaker grip strength, and increased sarcopenia risk, with supportive evidence from UK Biobank proteomic and Mendelian randomization analyses [49]. Circulating PBX1 has also emerged as a candidate biomarker associated with preserved muscle mass, gait speed, and handgrip strength, with alanine-related metabolites partially mediating its relationship with appendicular skeletal muscle mass [50]. In addition to inflammatory and mitochondrial biomarkers, emerging evidence suggests that lower serum uric acid concentrations are consistently associated with sarcopenia, whereas higher physiological uric acid levels correlate with greater skeletal muscle mass and handgrip strength. A recent meta-analysis including 16 studies further supports serum uric acid as a potential complementary biomarker for sarcopenia risk assessment, although its clinical interpretation should consider the complex balance between antioxidant protection and cardiometabolic risk [51]. Recent translational evidence combining in vitro, animal, and human data identified elevated circulating ceramides C18:0 and C24:1 as potential lipid biomarkers of sarcopenia, linking altered sphingolipid metabolism to oxidative stress, impaired myogenesis, and muscle dysfunction [52].

Artificial intelligence (AI) and machine learning approaches are increasingly being investigated to integrate clinical, imaging, and biomarker data into predictive models for sarcopenia diagnosis and prognosis. These computational approaches may help identify individualized biomarker patterns and improve precision medicine strategies in geriatric populations.

Biomarker research also has important therapeutic implications. Identification of central mechanistic pathways involved in sarcopenia may facilitate development of targeted interventions. For example:

- Mitochondrial-targeted therapies may reduce oxidative stress and improve bioenergetics;
- Anti-inflammatory therapies may attenuate chronic catabolic signaling;
- Senolytic therapies may eliminate senescent cells and reduce SASP burden;
- Neuromuscular-targeted interventions may preserve motor unit integrity;
- Anabolic therapies may improve protein synthesis and muscle regeneration.

Resistance exercise and nutritional interventions remain the cornerstone of sarcopenia management. However, biomarker-guided therapeutic strategies may eventually enable individualized treatment selection and monitoring of therapeutic response.

Despite considerable progress, several limitations remain before biomarker implementation in routine clinical practice becomes feasible. Many studies remain cross-sectional, biomarker assays lack standardization, and significant variability exists between populations and diagnostic criteria. Large longitudinal studies are therefore needed to validate biomarker combinations and establish clinically relevant cutoff values.

Importantly, future research should increasingly focus on identifying biomarkers associated with the earliest stages of sarcopenia. Early biological alterations occurring before significant muscle mass decline may represent the optimal therapeutic window for preventive interventions.

Collectively, current evidence supports the concept that integrated biomarker approaches combining mitochondrial, inflammatory, senescence-associated, neuromuscular, and anabolic pathways may substantially improve early diagnosis, prognosis, and therapeutic management of sarcopenia.

**Figure 9:**
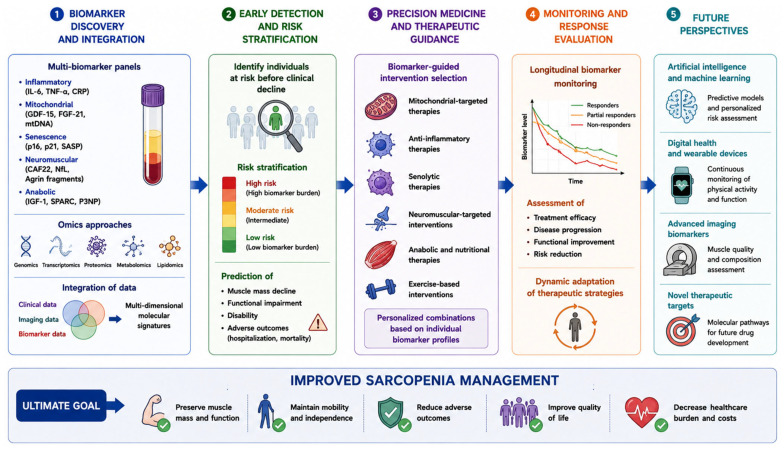
From Early Mechanisms to Clinical Biomarkers and Precision Medicine in Sarcopenia. This figure illustrates the integrated continuum linking the biological mechanisms underlying sarcopenia—including mitochondrial dysfunction, inflammaging, cellular senescence, neuromuscular dysfunction, and anabolic resistance—to the emergence of circulating biomarkers and their translation into clinical applications. It further highlights how multi-omics technologies, artificial intelligence, and longitudinal biomarker monitoring may facilitate early diagnosis, risk stratification, therapeutic monitoring, and personalized precision medicine strategies for sarcopenia. Concept conceived and initially sketched by the authors. Graphical rendering generated using OpenAI ChatGPT (GPT-5) and finalized in Microsoft PowerPoint by the authors. Abbreviations: IL-6: interleukin-6; TNF-α: tumor necrosis factor-alpha; CRP: C-reactive protein; GDF-15: growth differentiation factor 15; FGF-21: fibroblast growth factor 21; mtDNA: mitochondrial DNA; SASP: senescence-associated secretory phenotype; CAF22: C-terminal agrin fragment 22 kDa; NfL: neurofilament light chain; IGF-1: insulin-like growth factor-1; SPARC: secreted protein acidic and rich in cysteine; P3NP: procollagen type III N-terminal peptide.

The future perspectives and clinical applications of sarcopenia biomarkers are summarized in Figure 9. Currently available and emerging biomarkers, together with their potential clinical applications, are summarized in Table 8.

Although several candidate biomarkers have demonstrated promising associations with sarcopenia, their level of clinical validation remains highly variable. Some biomarkers have been evaluated in multiple human cohorts, whereas others are currently supported primarily by mechanistic or experimental studies. To facilitate interpretation of the current evidence and distinguish biomarkers according to their degree of clinical maturity, a critical appraisal of the principal candidate biomarkers is presented in Table 9.

While numerous candidate biomarkers have been investigated, their level of clinical validation differs substantially. Some biomarkers have been evaluated in multiple human sarcopenia cohorts, whereas others are currently supported mainly by mechanistic or experimental studies. To facilitate interpretation of the current evidence, the principal candidate biomarkers are critically appraised according to their level of human evidence, analytical characteristics, reproducibility, specificity, and clinical maturity (Table 9).

Overall, inflammatory biomarkers such as IL-6, TNF-α, and CRP have accumulated substantial clinical evidence but remain limited by poor disease specificity. In contrast, biomarkers such as GDF-15, CAF22, and FGF-21 appear more closely related to the underlying mechanisms of sarcopenia but still require additional longitudinal validation before routine clinical implementation. Emerging technologies, including multi-biomarker panels, omics approaches, artificial intelligence, and digital monitoring, remain promising research tools but require further standardization and prospective validation before widespread clinical adoption.

## 10. Limitations

Several limitations of the present review should be acknowledged.

First, although a structured literature search based on predefined search strategies was conducted and a PRISMA-inspired flow diagram was included to improve transparency, this work remains a narrative review rather than a formal systematic review. Consequently, some degree of study selection bias cannot be completely excluded.

Second, only English-language articles indexed in PubMed were considered. Relevant studies published in other languages or indexed in additional databases may therefore have been missed.

Third, no formal assessment of risk of bias or certainty of evidence was performed because the primary objective of this review was to provide an integrated mechanistic overview rather than a quantitative synthesis of clinical outcomes. Likewise, no meta-analysis was undertaken owing to the considerable heterogeneity of study designs, patient populations, biomarker assays, analytical methods, and outcome measures across the available literature.

Another important limitation concerns the current state of biomarker research itself. Although several circulating biomarkers—including GDF-15, FGF-21, IL-6, TNF-α, CAF22, IGF-1, and myostatin—have demonstrated promising associations with sarcopenia, many remain insufficiently validated for routine clinical implementation. Their concentrations are often influenced by age, sex, obesity, chronic kidney disease, cardiovascular disease, diabetes, cancer, nutritional status, and other age-related conditions, thereby limiting their specificity for skeletal muscle degeneration.

Furthermore, substantial methodological variability exists among published studies regarding sarcopenia definitions (EWGSOP2, AWGS, SDOC), laboratory assays, biological samples, and analytical protocols, making direct comparison between studies challenging. Finally, several biological pathways discussed in this review—particularly mitochondrial dysfunction, cellular senescence, and anabolic resistance—are currently supported predominantly by mechanistic or experimental evidence, whereas robust validation in large prospective human cohorts remains limited.

Despite these limitations, the present review provides an updated and integrated overview of the biological mechanisms underlying sarcopenia while critically summarizing the current evidence supporting candidate biomarkers with the greatest potential for future clinical translation.

## 11. Future Directions Toward Clinical Translation

Although remarkable progress has been achieved in understanding the biological mechanisms underlying sarcopenia, translating biomarker research into routine clinical practice remains a major challenge. Future investigations should move beyond the search for individual biomarkers and instead focus on integrated biological signatures capable of capturing the multifactorial nature of the disease.

One of the highest priorities should be the establishment of large multicenter prospective longitudinal cohorts using harmonized diagnostic criteria such as EWGSOP2, AWGS, and SDOC. Such studies are essential to determine the temporal sequence of biological alterations during sarcopenia development, identify biomarkers that precede measurable muscle loss, and validate their diagnostic and prognostic performance across diverse populations.

Future research should also prioritize multi-biomarker approaches rather than isolated circulating markers. Given the complex interactions between mitochondrial dysfunction, inflammaging, cellular senescence, neuromuscular degeneration, and anabolic resistance, combinations of complementary biomarkers—including GDF-15, FGF-21, IL-6, TNF-α, CAF22, IGF-1, myostatin, and emerging metabolomic signatures—are likely to provide substantially greater diagnostic accuracy than any single biomarker alone. These integrated panels should be evaluated together with muscle imaging, body composition measurements, handgrip strength, gait speed, and digital mobility assessments to improve patient phenotyping and risk stratification.

Rapid advances in multi-omics technologies are expected to transform biomarker discovery. Transcriptomics, proteomics, metabolomics, lipidomics, epigenomics, and single-cell sequencing now offer unprecedented opportunities to identify molecular networks involved in skeletal muscle ageing. Rather than focusing on isolated molecular pathways, future studies should integrate these complementary datasets to characterize distinct biological phenotypes of sarcopenia and identify novel therapeutic targets.

Artificial intelligence and machine-learning approaches are also likely to play an increasingly important role. By integrating clinical characteristics, imaging findings, functional assessments, wearable-device data, and complex molecular signatures, AI-based predictive models may facilitate earlier diagnosis, individualized risk prediction, therapeutic monitoring, and personalized treatment selection.

Finally, successful clinical translation will require international standardization of laboratory assays, pre-analytical procedures, reference intervals, and clinically meaningful cutoff values. Biomarker-guided interventional trials should determine whether early identification of high-risk individuals can improve therapeutic decision-making and whether biomarker-guided interventions—including resistance exercise, nutritional supplementation, mitochondrial-targeted therapies, anti-inflammatory approaches, and future senolytic strategies—translate into improved clinical outcomes. Such studies will represent a critical step toward precision medicine for sarcopenia.

## 12. Conclusions

Sarcopenia is a complex and multifactorial age-related disorder characterized not only by progressive loss of skeletal muscle mass and strength but also by profound biological alterations involving mitochondrial dysfunction, chronic inflammation, cellular senescence, neuromuscular degeneration, and anabolic resistance.

Current evidence increasingly supports the hypothesis that mitochondrial dysfunction may represent an upstream pathogenic event capable of initiating interconnected pathological cascades involving oxidative stress, inflammaging, senescence activation, denervation, and impaired muscle remodeling. These mechanisms do not operate independently but rather interact dynamically through reciprocal molecular cross-talk that progressively accelerates skeletal muscle degeneration.

Several circulating and molecular biomarkers have emerged as promising tools for improving understanding of sarcopenia pathogenesis and facilitating early diagnosis. Among them, GDF-15, FGF-21, IL-6, TNF-α, CAF22, p16INK4a, p21/CDKN1A, IGF-1, and myostatin appear particularly relevant because they reflect complementary biological pathways involved in disease progression.

Importantly, no single biomarker currently demonstrates sufficient diagnostic specificity or sensitivity for routine clinical use. Instead, integrative multi-biomarker approaches combining mitochondrial, inflammatory, senescence-associated, neuromuscular, and anabolic markers may provide more accurate characterization of sarcopenia phenotypes and improve risk stratification.

Emerging technologies including metabolomics, transcriptomics, proteomics, artificial intelligence, and digital biomarker monitoring may further transform future sarcopenia research and facilitate precision medicine strategies. Recent transcriptomic studies have identified novel cuproptosis-related genes (PDHA1, DLAT, PDHB and NDUFC1) as potential diagnostic biomarkers and therapeutic targets, illustrating the growing contribution of omics-based approaches to sarcopenia research [53]

Collectively, this review supports the concept that sarcopenia should be considered a systemic network disease driven by interconnected biological mechanisms rather than an isolated consequence of aging alone. Improved understanding of these pathways may facilitate development of early diagnostic strategies and novel therapeutic interventions targeting multiple mechanisms simultaneously.

Future advances will likely rely on integrated multi-biomarker signatures combined with artificial intelligence and multi-omics approaches, enabling earlier diagnosis, improved patient stratification, and personalized therapeutic interventions for sarcopenia.

## Figures and Tables

**Table 1:** Major biomarker categories involved in sarcopenia.

Category	Main Biomarkers	Main Biological Role	Variation in Sarcopenia	Clinical Significance	Representative References
Mitochondrial biomarkers	GDF-15, FGF-21, MMA, mtDNA	Mitochondrial stress, impaired energy metabolism, oxidative phosphorylation dysfunction	↑ Increased	Frailty, low muscle mass, mortality risk	Joseph et al., 2012 [5]; Conte et al., 2020 [9]; Tezze et al., 2017 [10]
Oxidative stress biomarkers	ROS, 8-OHdG, MDA	Oxidative damage to proteins, lipids, and DNA	↑ Increased	Cellular damage and muscle degeneration	Fulle et al., 2004 [11]; Joseph et al., 2012 [5]
Inflammatory biomarkers	IL-6, TNF-α, CRP	Chronic low-grade inflammation (“inflammaging”)	↑ Increased	Muscle catabolism and physical decline	Ferrucci et al., 2002 [12]; Bian et al., 2017 [13]
Senescence biomarkers	p16INK4a, p21/CDKN1A, SASP factors	Cell-cycle arrest and senescence-associated secretory phenotype	↑ Increased	Reduced regenerative capacity	Sousa-Victor et al., 2014 [14]; Childs et al., 2015 [15]; Baker et al., 2016 [16]
Neuromuscular biomarkers	CAF22, Agrin, NfL, BDNF	Neuromuscular junction integrity and neuronal function	CAF22/NfL ↑; Agrin/BDNF ↓	Muscle weakness and denervation	Drey et al., 2013 [17]; Jang & Van Remmen, 2011 [18]; Hepple & Rice, 2016 [19]
Anabolic/muscle biomarkers	IGF-1, Myostatin, P3NP, SPARC	Muscle protein synthesis and remodeling	IGF-1 ↓; Myostatin ↑	Muscle atrophy and impaired regeneration	Breen & Phillips, 2011 [20]; Li et al., 2005 [21]
Metabolomic biomarkers	Proline, Alanine, Tryptophan, BCAA	Amino acid metabolism and muscle energetics	Altered profiles	Early metabolic dysregulation	Calvani et al., 2015 [3]; Zhu et al., 2026 [7]
Functional biomarkers	Handgrip strength, gait speed, SPPB	Physical performance assessment	↓ Decreased	Diagnosis and severity assessment	Cruz-Jentoft et al., 2019 [1]; Larsson et al., 2019 [4]

Abbreviations: GDF-15: Growth Differentiation Factor-15; FGF-21: Fibroblast Growth Factor-21; MMA: methylmalonic acid; mtDNA: mitochondrial DNA; ROS: reactive oxygen species; 8-OHdG: 8-hydroxy-2′-deoxyguanosine; MDA: malondialdehyde; IL-6: interleukin-6; TNF-α: tumor necrosis factor-alpha; CRP: C-reactive protein; p16INK4a: cyclin-dependent kinase inhibitor 2A; p21/CDKN1A: cyclin-dependent kinase inhibitor 1A; SASP: senescence-associated secretory phenotype; CAF22: C-terminal agrin fragment; NfL: neurofilament light chain; BDNF: brain-derived neurotrophic factor; IGF-1: insulin-like growth factor-1; P3NP: procollagen type III N-terminal peptide; SPARC: secreted protein acidic and rich in cysteine; BCAA: branched-chain amino acids; SPPB: Short Physical Performance Battery.

**Table 2:** Major biomarkers involved in mitochondrial dysfunction and oxidative stress in sarcopenia.

Biomarker	Biological Role	Mechanism Involved	Variation in Sarcopenia	Clinical Significance	Representative References
GDF-15	Stress-responsive cytokine	Mitochondrial stress and inflammation	↑ Increased	Frailty, reduced muscle strength, mortality	Conte et al., 2020 [9]; Calvani et al., 2015 [3]
FGF-21	Metabolic regulator	Adaptive response to mitochondrial dysfunction	↑ Increased	Impaired muscle quality and physical performance	Tezze et al., 2017 [10]; Larsson et al., 2019 [4]
MMA	Metabolic intermediate	Defective mitochondrial metabolism	↑ Increased	Bioenergetic dysfunction	Calvani et al., 2015 [3]; Larsson et al., 2019 [4]
mtDNA	Mitochondrial DNA damage marker	Oxidative stress and mitochondrial injury	↑ Increased	Inflammaging and cellular damage	Joseph et al., 2012 [5]; Leduc-Gaudet et al., 2015 [24]
ROS	Oxidative stress mediator	Protein, lipid, and DNA oxidation	↑ Increased	Muscle degeneration and atrophy	Fulle et al., 2004 [11]; Joseph et al., 2012 [5]
CHCHD10	Mitochondrial homeostasis protein	Cristae organization and respiratory chain stability	Altered expression	Accelerated muscle aging	Ying et al., 2026 [27]; Guzman et al., 2026 [28]
SAMM50	Mitochondrial membrane protein	Mitochondrial morphology and quality control	Altered expression	Impaired mitochondrial integrity	Ying et al., 2026 [27]; Lu et al., 2022 [29]
MDH2	Tricarboxylic acid cycle enzyme	Oxidative phosphorylation and ATP production	Altered expression	Energetic deficiency	Ying et al., 2026 [27]; Brandão et al., 2021 [30]
PGC-1α	Mitochondrial biogenesis regulator	Oxidative metabolism and mitochondrial renewal	↓ Decreased	Reduced mitochondrial biogenesis	Short et al., 2005 [22]; Joseph et al., 2012 [5]
SIRT1	Cellular energy sensor	Mitophagy and oxidative stress regulation	↓ Decreased	Impaired mitochondrial adaptation	Tezze et al., 2017 [10]; Larsson et al., 2019 [4]

Abbreviations: GDF-15: Growth Differentiation Factor-15; FGF-21: Fibroblast Growth Factor-21; MMA: methylmalonic acid; mtDNA: mitochondrial DNA; ROS: reactive oxygen species; CHCHD10: coiled-coil-helix-coiled-coil-helix domain containing 10; SAMM50: sorting and assembly machinery component 50 homolog; MDH2: malate dehydrogenase 2; PGC-1α: peroxisome proliferator-activated receptor gamma coactivator 1-alpha; SIRT1: sirtuin 1.

**Table 3:** Major inflammatory biomarkers associated with sarcopenia.

Biomarker	Biological Role	Mechanism Involved	Variation in Sarcopenia	Clinical Significance	Representative References
IL-6	Pro-inflammatory cytokine	NF-κB activation and muscle catabolism	↑ Increased	Reduced muscle strength and frailty	Ferrucci et al., 2002 [12]; Bian et al., 2017 [13]; Calvani et al., 2015 [3]
TNF-α	Catabolic cytokine	Proteolysis and apoptosis	↑ Increased	Muscle wasting and functional decline	Li et al., 2005 [21]; Bian et al., 2017 [13]
CRP	Systemic inflammatory marker	Chronic low-grade inflammation	↑ Increased	Sarcopenia severity and mortality	Calvani et al., 2015 [3]; Ferrucci et al., 2002 [12]
NF-κB	Inflammatory transcription factor	Cytokine production and catabolism	↑ Activated	Chronic muscle inflammation	Li et al., 2005 [21]; Childs et al., 2015 [15]
cGAS-STING	Innate immune signaling pathway	mtDNA-induced inflammation	↑ Increased	Inflammaging and senescence	Childs et al., 2015 [15]; Baker et al., 2016 [16]
IL-1β	Pro-inflammatory cytokine	Inflammasome activation	↑ Increased	Muscle degeneration	Franceschi et al., 2000 [31]; Calvani et al., 2015 [3]
MCP-1	Chemokine	Immune cell recruitment	↑ Increased	Chronic inflammatory infiltration	Calvani et al., 2015 [3]; Childs et al., 2015 [15]
TGF-β	Fibrosis-associated cytokine	Fibrosis and impaired regeneration	↑ Increased	Muscle remodeling defects	Li et al., 2005 [21]; Larsson et al., 2019 [4]

Abbreviations: IL-6: interleukin-6; NF-κB: Nuclear factor kappa B; TNF-α: tumor necrosis factor-alpha; CRP: C-reactive protein; NF-κB: nuclear factor kappa B; cGAS: cyclic GMP-AMP synthase; STING: stimulator of interferon genes; mtDNA: mitochondrial DNA; IL-1β: interleukin-1 beta; MCP-1: monocyte chemoattractant protein-1; TGF-β: transforming growth factor-beta.

**Table 4:** Major senescence-associated biomarkers implicated in sarcopenia.

Biomarker	Biological Role	Mechanism Involved	Variation in Sarcopenia	Clinical Significance	Representative References
p16INK4a	Cyclin-dependent kinase inhibitor	Cellular senescence induction	↑ Increased	Stem-cell exhaustion and impaired regeneration	Baker et al., 2016 [16]; Childs et al., 2015 [15]; Campisi & d’Adda di Fagagna, 2007 [34]
p21/CDKN1A	Cell-cycle arrest mediator	DNA damage response and senescence	↑ Increased	Muscle atrophy and regenerative failure	Campisi & d’Adda di Fagagna, 2007 [34]; Childs et al., 2015 [15]; Sousa-Victor et al., 2014 [14]
SASP factors	Senescence-associated secretory phenotype	Chronic inflammatory amplification	↑ Increased	Inflammaging and muscle degeneration	Coppé et al., 2010 [35]; Childs et al., 2015 [15]
SA-β-gal	Senescence-associated lysosomal enzyme	Cellular aging marker	↑ Increased	Identification of senescent cells	Campisi & d’Adda di Fagagna, 2007 [34]; Childs et al., 2015 [15]
γH2AX	DNA damage marker	DNA repair activation	↑ Increased	Oxidative stress-induced senescence	Campisi & d’Adda di Fagagna, 2007 [34]; Childs et al., 2015 [15]
p53	Stress-response transcription factor	Apoptosis and senescence regulation	↑ Activated	Muscle degeneration and aging	Campisi & d’Adda di Fagagna, 2007 [34]; Baker et al., 2016 [16]
IL-6	SASP-associated cytokine	Chronic inflammation and catabolism	↑ Increased	Physical decline and frailty	Coppé et al., 2010 [35]; Ferrucci et al., 2002 [12]; Bian et al., 2017 [13]
TNF-α	Pro-inflammatory cytokine	Muscle proteolysis and inflammation	↑ Increased	Muscle wasting	Li et al., 2005 [21]; Bian et al., 2017 [13]
TGF-β	Fibrosis-associated SASP factor	Fibrosis and tissue remodeling	↑ Increased	Impaired muscle quality	Childs et al., 2015 [15]; Larsson et al., 2019 [4]
MMPs	Matrix remodeling enzymes	Extracellular matrix degradation	↑ Increased	Tissue remodeling and fibrosis	Coppé et al., 2010 [35]; Childs et al., 2015 [15]
MCP-1	Chemokine	Recruitment of inflammatory cells	↑ Increased	Chronic inflammatory infiltration	Childs et al., 2015 [15]; Calvani et al., 2015 [3]
VEGF	Growth factor	Angiogenesis and SASP signaling	Altered expression	Vascular remodeling	Childs et al., 2015 [15]; Larsson et al., 2019 [4]

Abbreviations: p16INK4a: cyclin-dependent kinase inhibitor 2A; p21/CDKN1A: cyclin-dependent kinase inhibitor 1A; SASP: senescence-associated secretory phenotype; SA-β-gal: senescence-associated beta-galactosidase; γH2AX: phosphorylated histone H2AX; p53: Tumor protein p53; IL-6: interleukin-6; TNF-α: tumor necrosis factor-alpha; TGF-β: transforming growth factor-beta; MMPs: matrix metalloproteinases; MCP-1: monocyte chemoattractant protein-1; VEGF: vascular endothelial growth factor.

**Table 5:** Major neuromuscular junction biomarkers associated with sarcopenia.

Biomarker	Biological Role	Mechanism Involved	Variation in Sarcopenia	Clinical Significance	Representative References
CAF22	Agrin cleavage product	NMJ degradation and denervation	↑ Increased	Muscle weakness and frailty	Drey et al., 2013 [17]; Calvani et al., 2015 [3]
Agrin	NMJ stabilizing protein	Acetylcholine receptor clustering	↓ Decreased	NMJ instability	Drey et al., 2013 [17]; Jang & Van Remmen, 2011 [18]
NfL	Axonal injury biomarker	Neurodegeneration and denervation	↑ Increased	Motor neuron dysfunction	Calvani et al., 2015 [3]; Larsson et al., 2019 [4]
BDNF	Neurotrophic factor	Motor neuron survival and regeneration	↓ Decreased	Impaired neuromuscular maintenance	Hepple & Rice, 2016 [19]; Larsson et al., 2019 [4]
AChRs	Synaptic transmission	NMJ signaling dysfunction	Altered expression	Reduced muscle activation	Jang & Van Remmen, 2011 [18]; Hepple & Rice, 2016 [19]
MuSK	NMJ signaling protein	Synaptic organization and maintenance	Altered expression	NMJ fragmentation	Jang & Van Remmen, 2011 [18]; Hepple & Rice, 2016 [19]
LRP4	Agrin receptor	NMJ stabilization	Altered expression	Synaptic destabilization	Jang & Van Remmen, 2011[18]; Hepple & Rice, 2016[19]
S100B	Neuroinflammatory marker	Glial activation and denervation	↑ Increased	Neuromuscular injury	Hepple & Rice, 2016 [19]
SYP	Synaptic vesicle protein	Neurotransmission integrity	↓ Decreased	Impaired synaptic function	Jang & Van Remmen, 2011 [18]; Larsson et al., 2019 [4]
NCAM	Neural adhesion molecule	Reinnervation and muscle repair	↑ Increased	Denervation marker	Hepple & Rice, 2016 [19]; Larsson et al., 2019 [4]

Abbreviations: CAF22: C-terminal agrin fragment; NMJ: neuromuscular junction; NfL: neurofilament light chain; BDNF: brain-derived neurotrophic factor; AChRs: acetylcholine receptors; MuSK: muscle-specific kinase; LRP4: low-density lipoprotein receptor-related protein 4; S100B: S100 calcium-binding protein B; SYP: Synaptophysin; NCAM: neural cell adhesion molecule.

**Table 6:** Major biomarkers associated with anabolic resistance and muscle remodeling in sarcopenia.

Biomarker	Biological Role	Mechanism Involved	Variation in Sarcopenia	Clinical Significance	Representative References
IGF-1	Anabolic growth factor	Akt/mTOR activation and protein synthesis	↓ Decreased	Reduced muscle mass and regeneration	Breen & Phillips, 2011 [20]; Calvani et al., 2015 [3]
Myostatin	Negative regulator of muscle growth	Inhibition of myogenesis and protein synthesis	↑ Increased	Muscle atrophy and weakness	Calvani et al., 2015 [3]; Larsson et al., 2019 [4]
Akt	Anabolic signaling kinase	Protein synthesis regulation	↓ Reduced activation	Anabolic resistance	Breen & Phillips, 2011 [20]; Larsson et al., 2019 [4]
mTOR	Protein synthesis regulator	Muscle hypertrophy signaling	↓ Reduced activation	Impaired muscle remodeling	Breen & Phillips, 2011 [20]; Larsson et al., 2019 [4]
MuRF1	Ubiquitin ligase	Proteolysis activation	↑ Increased	Muscle protein degradation	Li et al., 2005 [21]; Larsson et al., 2019 [4]
Atrogin-1	Muscle-specific ubiquitin ligase	Catabolic signaling	↑ Increased	Skeletal muscle atrophy	Li et al., 2005 [21]; Larsson et al., 2019 [4]
P3NP	Collagen turnover marker	Muscle remodeling	Altered levels	Muscle repair and fibrosis	Calvani et al., 2015 [3]; Larsson et al., 2019 [4]
SPARC	Extracellular matrix glycoprotein	Muscle adaptation and remodeling	Altered expression	Regenerative dysfunction	Calvani et al., 2015 [3]; Larsson et al., 2019 [4]
FOXO transcription factors	Catabolic regulators	Proteolysis and atrophy pathways	↑ Activated	Muscle wasting	Li et al., 2005 [21]; Larsson et al., 2019 [4]
MyoD	Myogenic regulatory factor	Muscle regeneration	↓ Decreased	Impaired satellite cell activation	Sousa-Victor et al., 2014 [14]; Larsson et al., 2019 [4]

Abbreviations: IGF-1: insulin-like growth factor-1; mTOR: mammalian target of rapamycin; Akt: Protein kinase B; MuRF1: muscle RING-finger protein-1; P3NP: procollagen type III N-terminal peptide; SPARC: secreted protein acidic and rich in cysteine; FOXO: forkhead box O transcription factors; MyoD: myogenic differentiation factor.

**Table 7:** Mechanistic interactions between major biomarker pathways involved in sarcopenia.

Primary Mechanism	Secondary Pathway Activated	Main Biomarkers Involved	Consequence on Skeletal Muscle
Mitochondrial dysfunction	Oxidative stress	GDF-15, FGF-21, ROS, mtDNA	ATP depletion and bioenergetic failure
Oxidative stress	Inflammatory activation	NF-κB, IL-6, TNF-α	Chronic catabolism
Inflammation	Cellular senescence	p16INK4a, p21/CDKN1A, SASP factors	Regenerative failure
Senescence	Anabolic resistance	IGF-1 ↓, mTOR ↓	Reduced protein synthesis
Denervation	Muscle atrophy	CAF22, NfL, Agrin ↓	Muscle weakness
Anabolic resistance	Proteolysis activation	MuRF1, Atrogin-1	Muscle mass loss
Inflammaging	NMJ instability	IL-6, TNF-α, ROS	Motor unit degeneration
Mitochondrial dysfunction	Senescence induction	p53/p21/CDKN1A, cGAS-STING	Accelerated muscle aging

Abbreviations: GDF-15: Growth Differentiation Factor-15; FGF-21: Fibroblast Growth Factor-21; ROS: reactive oxygen species; mtDNA: mitochondrial DNA; NF-κB: nuclear factor kappa B; IL-6: interleukin-6; TNF-α: tumor necrosis factor-alpha; p16INK4a: Cyclin-dependent kinase inhibitor 2A (CDKN2A); p21/CDKN1A: Cyclin-dependent kinase inhibitor 1A; SASP: senescence-associated secretory phenotype; IGF-1: insulin-like growth factor-1; NMJ: neuromuscular junction; mTOR: mammalian target of rapamycin; CAF22: C-terminal agrin fragment; NfL: neurofilament light chain; MuRF1: muscle RING-finger protein-1; p53: Tumor protein p53; cGAS: cyclic GMP-AMP synthase.

**Table 8:** Validated versus emerging biomarkers in sarcopenia.

Biomarker Category	Validated Biomarkers	Emerging Biomarkers	Potential Clinical Application
Inflammatory biomarkers	IL-6, CRP, TNF-α	cGAS-STING mediators	Risk stratification and prognosis
Mitochondrial biomarkers	GDF-15	FGF-21, mtDNA, MMA	Early detection of mitochondrial dysfunction
Senescence biomarkers	p16INK4a, p21/CDKN1A	SASP profiles, γH2AX	Biological aging assessment
Neuromuscular biomarkers	CAF22	NfL, Agrin fragments	Detection of denervation
Anabolic biomarkers	IGF-1	SPARC, P3NP	Evaluation of anabolic resistance
Functional biomarkers	Handgrip strength, gait speed	Digital mobility biomarkers	Functional monitoring
Omics biomarkers	—	Metabolomics and transcriptomics signatures	Precision medicine approaches

Abbreviations: IL-6: interleukin-6; CRP: C-reactive protein; TNF-α: tumor necrosis factor-alpha; cGAS: cyclic GMP-AMP synthase; GDF-15: Growth Differentiation Factor-15; FGF-21: Fibroblast Growth Factor-21; mtDNA: mitochondrial DNA; MMA: methylmalonic acid; p16INK4a: cyclin-dependent kinase inhibitor 2A; p21/CDKN1A: cyclin-dependent kinase inhibitor 1A; IGF-1: insulin-like growth factor-1; SASP: senescence-associated secretory phenotype; γH2AX: phosphorylated histone H2AX; CAF22: C-terminal agrin fragment; NfL: neurofilament light chain; SPARC: secreted protein acidic and rich in cysteine; P3NP: procollagen type III N-terminal peptide.

**Table 9:** Critical appraisal of candidate biomarkers for sarcopenia.

Bio-Marker	Main Biological Pathway	Sample Type	Detection Method	Human Evidence	Reproducibility	Specificity	Main Confounders	Clinical Maturity
GDF-15	Mitochondrial dysfunction/cellular stress	Serum/Plasma	ELISA	Multiple cohorts	Moderate-High	Low-Moderate	Age, CKD, cancer, CVD	Promising
FGF-21	Mitochondrial dysfunction	Serum/Plasma	ELISA	Several cohorts	Moderate	Low	Diabetes, obesity, liver disease	Emerging
IL-6	Chronic inflammation	Serum/Plasma	ELISA	Multiple cohorts	High	Low	Inflammatory diseases	Supportive
TNF-α	Chronic inflammation	Serum/Plasma	ELISA	Multiple cohorts	Moderate-High	Low	Inflammatory diseases	Supportive
CRP	Systemic inflammation	Serum/Plasma	Immunoassay	Multiple cohorts	High	Very low	Acute/chronic inflammation	Supportive
CAF22	Neuromuscular degeneration	Serum	ELISA	Several studies	Moderate	Moderate	Neuromuscular disorders	Promising
NfL	Axonal degeneration	Serum/Plasma	Simoa	Limited	Moderate	Low	Neurodegenerative diseases	Emerging
p16INK4a	Cellular senescence	Muscle biopsy	qPCR/IHC	Limited	Moderate	Moderate	Aging	Experimental
p21/CDKN1A	Cellular senescence	Muscle biopsy	qPCR/IHC	Limited	Moderate	Moderate	Cellular stress	Experimental
IGF-1	Anabolic signaling	Serum	Immunoassay	Multiple cohorts	Moderate-High	Low	Nutrition/endocrine	Supportive
Myostatin	Muscle growth inhibition	Serum/Plasma	ELISA	Several cohorts	Moderate	Low-Moderate	Activity, obesity	Emerging
MuRF1	Proteolysis	Muscle biopsy	qPCR/Western blot	Experimental	Low	Low	Disuse, cachexia	Experimental
Atrogin-1	Proteolysis	Muscle biopsy	qPCR/Western blot	Experimental	Low	Low	Disuse	Experimental
FOXO	Catabolic transcription	Muscle biopsy	qPCR/Western blot	Experimental	Low	Low	Metabolic stress	Experimental
P3NP	Muscle remodeling	Serum	Immunoassay	Limited	Moderate	Low	Fibrosis	Emerging
SPARC	Extracellular matrix remodeling	Serum/Muscle	ELISA/qPCR	Limited	Low-Moderate	Low	Exercise	Experimental
MMA	Mitochondrial metabolism	Serum/Plasma	LC-MS/MS	Limited	Moderate	Low	B12 deficiency, renal disease	Emerging
mtDNA	Mitochondrial damage	Plasma/Serum	qPCR	Limited	Moderate	Low-Moderate	Tissue injury	Experimental

Abbreviations: GDF-15: Growth Differentiation Factor-15; ELISA: Enzyme-linked immunosorbent assay; CKD: Chronic kidney disease; CVD: Cardiovascular disease; FGF-21: Fibroblast Growth Factor-21; IL-6: interleukin-6; TNF-α: tumor necrosis factor-alpha; CRP: C-reactive protein; CAF22: C-terminal agrin fragment; NfL: neurofilament light chain; Simoa: Single-molecule array; p16INK4a: cyclin-dependent kinase inhibitor 2A; qPCR: Quantitative polymerase chain reaction; IHC: Immunohistochemistry; p21/CDKN1A: cyclin-dependent kinase inhibitor 1A; IGF-1: insulin-like growth factor-1; MuRF1: muscle RING-finger protein-1; FOXO: forkhead box O transcription factors; P3NP: procollagen type III N-terminal peptide; SPARC: secreted protein acidic and rich in cysteine; MMA: methylmalonic acid; LC-MS/MS: Liquid chromatography–tandem mass spectrometry; mtDNA: mitochondrial DNA.

## Data Availability

No new data were created or analyzed in this study. Data sharing is not applicable to this article.

## References

[1] Cruz-Jentoft A.J., Bahat G., Bauer J., Boirie Y., Bruyère O., Cederholm T., Cooper C., Landi F., Rolland Y., Sayer A.A. (2019). Sarcopenia: Revised European consensus on definition and diagnosis. Age Ageing.

[2] Dent E., Morley J.E., Cruz-Jentoft A.J., Arai H., Kritchevsky S.B., Guralnik J., Bauer J.M., Pahor M., Clark B.C., Cesari M. (2018). International Clinical Practice Guidelines for Sarcopenia (ICFSR): Screening, Diagnosis and Management. J. Nutr. Health Aging.

[3] Calvani R., Marini F., Cesari M., Tosato M., Anker S.D., Von Haehling S., Miller R.R., Bernabei R., Landi F., Marzetti E. (2015). Biomarkers for physical frailty and sarcopenia: State of the science and future developments. J. Cachexia Sarcopenia Muscle.

[4] Larsson L., Degens H., Li M., Salviati L., Lee Y.I., Thompson W., Kirkland J.L., Sandri M. (2019). Sarcopenia: Aging-Related Loss of Muscle Mass and Function. Physiol. Rev..

[5] Joseph A., Adhihetty P.J., Buford T.W., Wohlgemuth S.E., Lees H.A., Nguyen L.M., Aranda J.M., Sandesara B.D., Pahor M., Manini T.M. (2012). The impact of aging on mitochondrial function and biogenesis pathways in skeletal muscle of sedentary high- and low-functioning elderly individuals. Aging Cell.

[6] Martínez-Reyes I., Diebold L.P., Kong H., Schieber M., Huang H., Hensley C.T., Mehta M.M., Wang T., Santos J.H., Woychik R. (2016). TCA Cycle and Mitochondrial Membrane Potential Are Necessary for Diverse Biological Functions. Mol. Cell.

[7] Zhu J., Zhu T., Lai K., Hu C., Lv Z., Lai C., Xu Z., Su L. (2026). Mediation analysis of metabolic and inflammatory markers in the association between physical activity and musculoskeletal disease: Findings from NHANES 2013–2018. Eur. J. Appl. Physiol..

[8] Won C.W., Kim M., Shin H.E. (2025). From a Solitary Blood-Derived Biomarker to Combined Biomarkers of Sarcopenia: Experiences from the Korean Frailty and Aging Cohort Study. J. Gerontol. Ser. A.

[9] Conte M., Martucci M., Mosconi G., Chiariello A., Cappuccilli M., Totti V., Santoro A., Franceschi C., Salvioli S. (2020). GDF15 Plasma Level Is Inversely Associated with Level of Physical Activity and Correlates with Markers of Inflammation and Muscle Weakness. Front. Immunol..

[10] Tezze C., Romanello V., Desbats M.A., Fadini G.P., Albiero M., Favaro G., Ciciliot S., Soriano M.E., Morbidoni V., Cerqua C. (2017). Age-Associated Loss of OPA1 in Muscle Impacts Muscle Mass, Metabolic Homeostasis, Systemic Inflammation, and Epithelial Senescence. Cell Metab..

[11] Fulle S., Protasi F., Di Tano G., Pietrangelo T., Beltramin A., Boncompagni S., Vecchiet L., Fanò G. (2004). The contribution of reactive oxygen species to sarcopenia and muscle ageing. Exp. Gerontol..

[12] Ferrucci L., Penninx B.W.J.H., Volpato S., Harris T.B., Bandeen-Roche K., Balfour J., Leveille S.G., Fried L.P., Guralnik J.M. (2002). Change in Muscle Strength Explains Accelerated Decline of Physical Function in Older Women with High Interleukin-6 Serum Levels. J. Am. Geriatr. Soc..

[13] Bian A.-L., Hu H.-Y., Rong Y.-D., Wang J., Wang J.-X., Zhou X.-Z. (2017). A study on relationship between elderly sarcopenia and inflammatory factors IL-6 and TNF-α. Eur. J. Med. Res..

[14] Sousa-Victor P., Gutarra S., García-Prat L., Rodriguez-Ubreva J., Ortet L., Ruiz-Bonilla V., Jardí M., Ballestar E., González S., Serrano A.L. (2014). Geriatric muscle stem cells switch reversible quiescence into senescence. Nature.

[15] Childs B.G., Durik M., Baker D.J., van Deursen J.M. (2015). Cellular senescence in aging and age-related disease: From mechanisms to therapy. Nat. Med..

[16] Baker D.J., Childs B.G., Durik M., Wijers M.E., Sieben C.J., Zhong J., Saltness R.A., Jeganathan K.B., Verzosa G.C., Pezeshki A. (2016). Naturally occurring p16Ink4a-positive cells shorten healthy lifespan. Nature.

[17] Drey M., Sieber C., Bauer J., Uter W., Dahinden P., Fariello R., Vrijbloed J. (2013). C-terminal Agrin Fragment as a potential marker for sarcopenia caused by degeneration of the neuromuscular junction. Exp. Gerontol..

[18] Jang Y.C., Van Remmen H. (2011). Age-associated alterations of the neuromuscular junction. Exp. Gerontol..

[19] Hepple R.T., Rice C.L. (2016). Innervation and neuromuscular control in ageing skeletal muscle. J. Physiol..

[20] Breen L., Phillips S.M. (2011). Skeletal muscle protein metabolism in the elderly: Interventions to counteract the ‘anabolic resistance’ of ageing. Nutr. Metab..

[21] Li Y.-P., Chen Y., John J., Moylan J., Jin B., Mann D.L., Reid M.B. (2005). TNF-α acts via p38 MAPK to stimulate expression of the ubiquitin ligase atrogin1/MAFbx in skeletal muscle. FASEB J..

[22] Short K.R., Bigelow M.L., Kahl J., Singh R., Coenen-Schimke J., Raghavakaimal S., Nair K.S. (2005). Decline in skeletal muscle mitochondrial function with aging in humans. Proc. Natl. Acad. Sci. USA.

[23] Zhu H., Sun Q., Tang H., Chen Y., Tan K., Xu X., Wang S. (2023). A novel rat model of sarcopenic obesity based on aging and high-fat diet consumption. Biogerontology.

[24] Leduc-Gaudet J.-P., Picard M., Pelletier F.S.-J., Sgarioto N., Auger M.-J., Vallée J., Robitaille R., St-Pierre D.H., Gouspillou G. (2015). Mitochondrial morphology is altered in atrophied skeletal muscle of aged mice. Oncotarget.

[25] Profili N.I., Fiorillo E., Orrù V., Benelli M., Cucca F., Delitala A.P. (2026). Growth Differentiation Factor 15 and Physical Function Impairment in the SardiNIA Study. J. Clin. Med..

[26] Laosa O., Carretero A., Rodríguez-Mañas L., Carnicero J.A., Viña J., Gómez-Cabrera M.C., Angulo J., García-García F.J., El Assar M. (2026). Gender Differences in Circulating TFAM Levels Are Associated with Functional Impairment and Sarcopenia. Biomedicines.

[27] Ying H., Wang W., Huang L., Hong W., Yang L. (2026). Single-cell RNA sequencing and integrated bioinformatics reveal new mitochondrial biomarkers in sarcopenia. Front. Mol. Biosci..

[28] Guzman S.D., Fraczek P.M., Itsani K., Furati E.K., Juros D., Kenney G., Valdez G., Chakkalakal J.V., Aguilar C.A. (2026). Age-Associated Dysregulation of Postsynaptic Mitochondria Perturbs Reinnervation Kinetics. Aging Cell.

[29] Lu X., Gong Y., Hu W., Mao Y., Wang T., Sun Z., Su X., Fu G., Wang Y., Lai D. (2022). Ultrastructural and proteomic profiling of mitochondria-associated endoplasmic reticulum membranes reveal aging signatures in striated muscle. Cell Death Dis..

[30] Brandão S.R., Reis-Mendes A., Domingues P., Duarte J.A., Bastos M.L., Carvalho F., Ferreira R., Costa V.M. (2021). Exploring the aging effect of the anticancer drugs doxorubicin and mitoxantrone on cardiac mitochondrial proteome using a murine model. Toxicology.

[31] Franceschi C., Bonafè M., Valensin S. (2000). Human immunosenescence: The prevailing of innate immunity, the failing of clonotypic immunity, and the filling of immunological space. Vaccine.

[32] Ying L., Zhang Q., Yang Y.-M., Zhou J.-Y. (2022). A Combination of Serum Biomarkers in Elderly Patients with Sarcopenia: A Cross-Sectional Observational Study. Int. J. Endocrinol..

[33] Xie Y., Han Z., Chen Z., Ye X. (2026). Association of the neutrophil percentage-to-albumin ratio with sarcopenia in U.S. adults: Evidence from NHANES 2011–2018 and machine learning–based analyses. Exp. Gerontol..

[34] Campisi J., d’Adda di Fagagna F. (2007). Cellular senescence: When bad things happen to good cells. Nat. Rev. Mol. Cell Biol..

[35] Coppé J.-P., Desprez P.-Y., Krtolica A., Campisi J. (2010). The Senescence-Associated Secretory Phenotype: The Dark Side of Tumor Suppression. Annu. Rev. Pathol. Mech. Dis..

[36] Landi F., Calvani R., Lorenzi M., Martone A.M., Tosato M., Drey M., D’ANgelo E., Capoluongo E., Russo A., Bernabei R. (2016). Serum levels of C-terminal agrin fragment (CAF) are associated with sarcopenia in older multimorbid community-dwellers: Results from the ilSIRENTE study. Exp. Gerontol..

[37] Chen J., Cheng N., Liu Y., Xu T., Xi L., Wang C., Ouyang X. (2026). Agrin as a Stable Biomarker for Muscle Strength Decline in Elderly Sarcopenic Patients Associated with Neuromuscular Junction Dysfunction. Rejuvenation Res..

[38] Zhang C., Liu Y., Shen J., Zhang Y., Liu Y., Zhang K., Fan G., Liao J., Dai D., Zeng P. (2026). Brain-Derived Neurotrophic Factor, Sarcopenia and Digital Gait Characteristics in Older Adults: Insights into the Brain–Muscle Axis. J. Cachex Sarcopenia Muscle.

[39] Glass D.J. (2005). Skeletal muscle hypertrophy and atrophy signaling pathways. Int. J. Biochem. Cell Biol..

[40] McPherron A.C., Lawler A.M., Lee S.-J. (1997). Regulation of skeletal muscle mass in mice by a new TGF-p superfamily member. Nature.

[41] Bodine S.C., Latres E., Baumhueter S., Lai V.K.-M., Nunez L., Clarke B.A., Poueymirou W.T., Panaro F.J., Na E., Dharmarajan K. (2001). Identification of Ubiquitin Ligases Required for Skeletal Muscle Atrophy. Science.

[42] Burton A.N., Gharibzadeh S., Funnell M.P., Khunti K., Watson E.L., Salisu-Olatunji S.O., Shojaei F.S., Naderpour S., Hassam K.H., Wilkinson T.J. (2026). The diagnostic accuracy of blood biomarkers for sarcopenia, sarcopenic obesity, and osteosarcopenia: A meta-analysis. Arch. Gerontol. Geriatr..

[43] Engelmann S.U., Kasparbauer F., Pickl C., Del Giudice F., Haas M., Rinderknecht E., Siska P.J., Pichler R., Niessen C., Burger M. (2025). Interplay of IL-6, GDF-15 and Sarcopenia in Patients With Bladder Cancer Undergoing Radical Cystectomy and Its Implications on Survival. J. Cachex Sarcopenia Muscle.

[44] Damanti S., Sciorati C., De Lorenzo R., Avola A., Ruggiero M.P., Santoro S., Senini E., Messina M., Farina F., Festorazzi C. (2025). Circulating mitokines GDF-15 and FGF21 are associated with frailty, sarcopenia, and malnutrition in older adults: Evidence from the FRASNET study. Mech. Ageing Dev..

[45] Lapauw L., Vermeiren L., Vercauteren L., Amini N., Peeters L., Dalle S., Koppo K., Derrien M., Dupont J., Raes J. (2026). An exploratory multi-biomarker panel including fecal calprotectin, Brain-Derived Neurotrophic Factor, Fibroblast-Growth Factor-21 and irisin shows poor diagnostic accuracy for detecting probable sarcopenia in community-dwelling older persons. Aging Clin. Exp. Res..

[46] Tsai J., Wang S., Chang C., Chen C., Wen C., Chen G., Kuo C., Tseng Y.J., Chen C. (2022). Identification of traumatic acid as a potential plasma biomarker for sarcopenia using a metabolomics-based approach. J. Cachex Sarcopenia Muscle.

[47] Liu X., Li H., Zuo W., Wang G., Jiang J., Li Z., He X., Wang W., Yue Y., Zhang Y. (2026). Circulating immunometabolic markers associated with sarcopenia and muscle strength at high altitude: An integrated multi-omics study. J. Nutr. Health Aging.

[48] Lv X.-G., Liu K.-W., Tian C.-Y., Wang C., Jin X.-Y., Hu Y., Mao X.-Q., Zhang W.-W., Jing L.-P. (2026). Association between bile acid metabolism and sarcopenia in older adults. Clin. Chim. Acta.

[49] Kim J.Y., Jo Y., Park S.J., Baek J.Y., Jang G., Lee E., Sakong H., Kim S.J., Kim S.-J., Ryu D. (2026). Higher Circulating Kynurenine Levels Linked to Higher Risk of Sarcopenia in Older Adults: A Cohort Study and UK Biobank Analysis. Endocrinol. Metab..

[50] Meng X., Zhao Z., Mao S., Yu J., Liu Y., Yang X., Zhang X., Guo R., Yang S., Liang Z. (2026). Circulating PBX1 and age-related sarcopenia phenotypes: Metabolic mediation analyses in a community-based study. Eur. Geriatr. Med..

[51] He J., Hu F.B., Wang Y.B., Mei Y.B. (2024). Potential protective effects of increased serum uric acid concentration in sarcopenia: A meta-analysis and systematic review. Medicine.

[52] Park S.J., Baek J.Y., Wei S., Lee J.Y., Lee Y., Lee S., Jang I., Sakong H., Yoo H.J., Jung H. (2026). Elevated Circulating Ceramides 18:0 and 24:1 as a Risk Factor for Sarcopenia: In Vitro, Animal, and Clinical Evidence. J. Cachex Sarcopenia Muscle.

[53] Zhu Y., Chen X., Geng S., Li Q., Li Y., Yuan H., Jiang H. (2023). Identification of the cuproptosis-related hub genes and therapeutic agents for sarcopenia. Front. Genet..

